# Decadal scale phytoplankton species miniaturization in subtropical coastal waters

**DOI:** 10.1093/ismejo/wraf257

**Published:** 2025-11-20

**Authors:** Zhimeng Xu, Xiaodong Zhang, Mingjue Li, Wenzhao Liang, Yu Ma, Lixia Deng, Jiawei Chen, Kailin Liu, Hongbin Liu

**Affiliations:** Haide College, Ocean University of China, Qingdao, 266100, China; Department of Ocean Science, The Hong Kong University of Science and Technology, Clear Water Bay, Kowloon 999077, Hong Kong, China; Haide College, Ocean University of China, Qingdao, 266100, China; Department of Ocean Science, The Hong Kong University of Science and Technology, Clear Water Bay, Kowloon 999077, Hong Kong, China; College of Marine Life Science, Ocean University of China, Qingdao, 266003, China; Department of Ocean Science, The Hong Kong University of Science and Technology, Clear Water Bay, Kowloon 999077, Hong Kong, China; Department of Ocean Science, The Hong Kong University of Science and Technology, Clear Water Bay, Kowloon 999077, Hong Kong, China; College of the Environment & Ecology, Xiamen University, Xiamen, 361102, China; Department of Ocean Science, The Hong Kong University of Science and Technology, Clear Water Bay, Kowloon 999077, Hong Kong, China; CAS-HKUST Sanya Joint Laboratory of Marine Science Research, Key Laboratory of Tropical Marine Biotechnology of Hainan Province, Sanya Institute of Oceanology, SCSIO, Sanya, 572000, China

**Keywords:** phytoplankton miniaturization, coastal waters, global warming, interactive effects, environmental protection

## Abstract

Miniaturization, i.e. reduction in body size, happens in different organisms as an adaptation strategy under environmental stress such as warming. However, whether phytoplankton miniaturization occurs in coastal waters remains understudied due to complex environmental factors and strong spatiotemporal variability. Here, we comprehensively investigated the long-term changes in phytoplankton body size over 20 years in the coastal waters of Hong Kong through monthly sampling at 25 stations across the region. We employed a framework distinguishing two drivers of community miniaturization: (i) intraspecific size reduction (species miniaturization) and (ii) shifts in community composition toward a higher proportion of small species. At the species level, miniaturization was widespread, more in diatoms than dinoflagellates, primarily driven by temperature, supporting the temperature-size relation. In contrast, community-level miniaturization was negligible across most stations (except in a semi-closed bay), which was attributed to the decreased proportion of small species. This could be explained by the declined phosphate concentration which not only directly reduced the proportion of small species but also diminished the temperature sensitivity of phytoplankton community. Our findings provide multiscale insights into coastal phytoplankton miniaturization, with critical implications for food web dynamics and the biological carbon pump. Moreover, we highlight that anthropogenic nutrient reduction may significantly mitigate community-level phytoplankton miniaturization, though localized effects in semi-enclosed systems warrant further investigation.

## Introduction

Marine phytoplankton play fundamental roles in the global biogeochemical cycles and climate variability. Understanding how they respond to environmental changes is crucial for deciphering the complex two-way interaction between phytoplankton communities and the ocean environmental conditions [1]. As a “master trait”, body size governs multiple aspects of phytoplankton ecology, including resource acquisition (particularly nutrients), growth rate, swimming and sinking rates, competitive interaction, and trophic transfer efficiency [2]. Phytoplankton size holds crucial ecological significances across biological scales, influencing species adaptation, evolution, and community regime shift [3]. Investigating the spatiotemporal patterns of phytoplankton body size and their driving factors is therefore essential for understanding the micro- and macroecology of phytoplankton, improving marine ecosystem models, particularly trait-based approaches [4].

Previous studies on marine phytoplankton size have primarily focused on the community level, leaving intraspecific size trends largely unexplored [2]. Size fractionation by filtration (measuring abundance, biomass, or chlorophyll) is a widely used technique to investigate the size change of phytoplankton at the community level. Though this approach has shown a general shift toward smaller phytoplankton in warmer waters [5, 6], it fails to distinguish between species size change and exchange of species—two fundamentally different processes underlying community size structure. On the contrary, microscopy-based studies have provided more precise measurements of individual cell sizes, demonstrating how temperature and nutrients affect phytoplankton physiology in controlled experiments [7, 8]. However, these studies typically examined short-term responses (weeks to months) of selected species, limiting insights into long-term adaptation or community-level consequences.

The long-term trends (e.g. annual) in marine phytoplankton size are much less studied, with most studies relying on size fractionation and leaving species-level variations unclear [9–12]. One reason is due to the labor-intensive nature of phytoplankton identification and size measurement for a long-term continuous sampling. Additionally, seasonal variation in phytoplankton size can be pronounced, often masking the annual trend which may need decades to show the evolutionary adaptation. Particularly, community size of coastal phytoplankton could be frequently and largely changed by algal blooms, commonly formed by diatom and flagellate species. For instance, some dinoflagellate species can fall into the microplankton category, dramatically increasing the average phytoplankton community size during blooms [13, 14]. These periodical events can have great impacts on evaluating the annual trend in community size when the sampling efforts are insufficient or uneven across different seasons.

Marine phytoplankton sizes are regulated by not only physical factors (mainly temperature) but also nutrient (e.g. nitrate, phosphate, and silicate) dynamics and predation stress (e.g. from zooplankton) [2]. For temperature, three ecological rules stand for decades as hallmarks to state the negative correlations between body size and temperature. First, Bergmann’s rule, the ﻿oldest manifestations of a “biogeography of traits”, posits that warmer regions tend to harbor smaller species [15]. Second, the temperature–size rule (TSR) describes an inverse relationship between organism size and rearing temperature, primarily observed in ectotherms [16]. Third, James’ rule notes that, within a given species, populations in warmer environments typically exhibit smaller body sizes [17]. Further, researchers proposed applying Bergmann’s rule to species replacement patterns (e.g. within genera or higher taxonomy levels) and James’ rule to intraspecific size trends [18]. In contrast, the TSR, which argues that high temperature drives the ontogeny maturation of species at early life stage with a smaller body size, is strictly phenotypic without involving any species replacement. Therefore, the conceptual distinctions among the three rules are directly linked to biological scale, ranging from species (body size change due to self-adaptation, TSR) to community (interspecific replacement leading to community turnover, Bergmann’s rule) levels, each carrying distinct ecological implications on marine ecosystems. However, the application of these rules, with partitioning the contribution of different processes, has received little attention on phytoplankton. Besides, nutrients are also a key determinant of phytoplankton size. For instance, larger species require higher nutrient uptake fluxes to support cell division and can sustain greater biomass-specific production rates in nutrient-rich conditions than their smaller counterparts [19, 20]. Furthermore, the influence of temperature on cell size is largely mediated by nutrient levels [21, 22], highlighting the complex interactive effects of environmental factors on phytoplankton size structure.

Regulating mechanisms of coastal phytoplankton sizes are often obscured by high spatiotemporal heterogeneity and potential co-limiting environmental factors. Consequently, although numerous studies document phytoplankton miniaturization, the driving factors, or fundamental processes remain unclear [23–25]. Furthermore, strong co-variation among environmental parameters in coastal waters may impede the identification of primary driving factors of phytoplankton size [26, 27]. The interaction effects of these factors (e.g. between temperature and nutrients) on phytoplankton size, which have been well studied in manipulated micro- or mesocosm experiments with single or multiple species [7, 8, 21], are also lacking of reporting in field survey, especially at the community level.

The subtropical coastal waters of Hong Kong offer an ideal environment for studying phytoplankton. Temporally, subtropical monsoon influences create distinct patterns of phytoplankton communities between wet (summer) and dry (winter) seasons [28]. Spatially, sharp nutrient gradients emerge between the Pearl River-influenced, nutrient-rich western waters and the oligotrophic southern waters connected to the South China Sea [29]. Thanks to the routine monthly monitoring efforts from Environmental Protection Department (EPD) in Hong Kong, these unique hydrological characters have been well measured, recorded online, and frequently used to assess natural and anthropogenic impacts on phytoplankton communities [30, 31]. While warming and eutrophication effects in selected localized areas were well-documented [30, 32], government actions of environmental protection have been taken place since 1985 and made significant achievements on nutrient removal especially in the semi-closed region of Tolo Harbour [33]. However, the size variation of phytoplankton, especially the long-term trend, in response to these environmental changes have not been reported.

**Figure 1:**
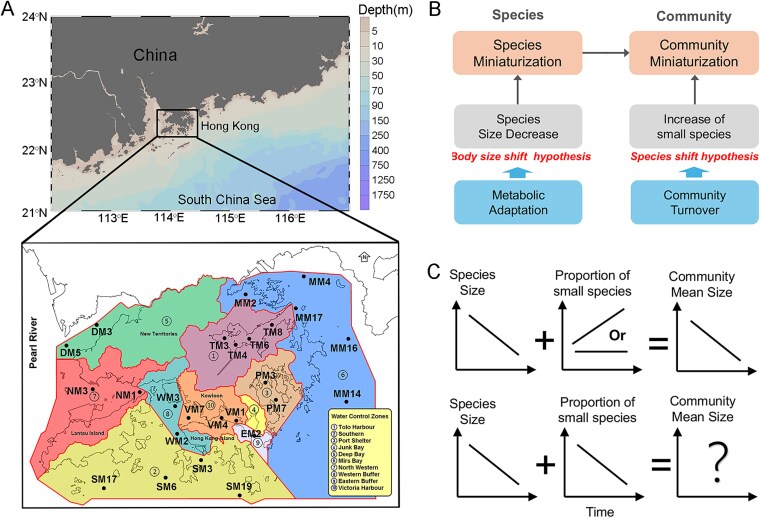
Sampling stations and conceptual framework of phytoplankton miniaturization. **(A)** Map showing 25 sampling stations in the coastal water of Hong Kong, categorized into 10 water control zones (WCZs); **(B)** analytical framework for phytoplankton size at both species and community levels. Community miniaturization results from species miniaturization (body size shift hypothesis) and increased proportion of small species (Small%) (species shift hypothesis); **(C)** two potential scenarios of community size change were proposed based on the combined efforts of species size and Small%: (i) community miniaturization occurs under decreasing species size and increasing/stable Small%; (ii) uncertain pattern of community mean size under the deceasing trends of both species size and Small%. As miniaturization was focused, increase of species size was not hypothesized here.

Here, using a 20-year dataset of phytoplankton communities (monthly collected from 25 stations covering the entire Hong Kong coastal region), along with 25 types of environmental parameters, we analyzed the phytoplankton size trends at both species and community levels and their primarily driving factors (Fig. 1A). We developed a conceptual framework partitioning community miniaturization into two components: species miniaturization (body size shift hypothesis) and increasing proportion of small species (Small%; species shift hypothesis) (Fig. 1B and C). We hypothesized that (i) phytoplankton in Hong Kong coastal waters are facing miniaturization at both species and community levels; (ii) temperature and nutrients play distinct regulatory roles on phytoplankton size; (iii) historical actions of environmental protection, such as nutrient removal, may have significant effects on phytoplankton miniaturization. Specifically, we aimed to (i) reveal the long-term phytoplankton size change at the species level, with the comparison among key phytoplankton groups; and (ii) identify the driving factors and processes regulating coastal phytoplankton size trends, with the application and testing of different ecological theories.

## Materials and Methods

### Study area

The study area encompassed 25 monitoring stations, covering the entire Hong Kong coastal waters (Fig. 1). According to the classification of EPD in Hong Kong, these stations are distributed across 10 water control zones (WCZs) with distinct hydrological characteristics (https://cd.epic.epd.gov.hk/EPICRIVER/marine/). For instance, the Deep Bay (DM3 and DM5) and Northwestern (NM1 and NM3) regions are heavily influenced by the seasonal discharge of freshwater from Pearl River. In contrast, Southern (e.g. SM6 and SM19) and Mirs Bay (e.g. MM6 and MM14) are more influenced by the relatively oligotrophic pelagic waters from the South China Sea. Semi-closed harbors, such as Tolo Harbour (TM3 to TM8), show greater impacts from land-based nutrient inputs and anthropogenic activities.

### Environmental factors

A comprehensive set of 25 environmental parameters were measured at each sampling station, including physicochemical properties (e.g. temperature, salinity, and dissolved oxygen), macronutrient concentrations (e.g. nitrogen, phosphate, and silicate) and biologically relevant indices (e.g. 5-day biochemical oxygen demand [BOD5] and chlorophyll *a*). Detailed information including abbreviations, units, detection limits, analytical methods and ecological implications is listed in the supplementary file (Table S1). Raw data is available from the EDP website (https://cd.epic.epd.gov.hk/EPICRIVER/marine/). For key environmental variables (temperature, dissolved inorganic nitrogen, phosphate, and N/P ratio), long-term trends (from 1986 to 2020) were analyzed using linear regression to determine annual trendlines and slopes for each station. The environmental factors displayed here were collected from 1986 to 2020, with the aim to reflect the efforts of environmental protection since in 1987 (EPD, Hong Kong).

### Sample collection, species identification, and community analysis

Phytoplankton were collected monthly for each station for 20 years from 2000 to 2020, generating a total of 6083 samples. In brief, seawater was collected from 1 m below the sea surface using either a 3 l van Dorn sampler or several 2.5 l Niskin bottles attached to a Sea-Bird SBE 32 carousel water sampler. All samples were fixed with 1.0% Lugol’s iodine solution *in situ* and stored in dark plastic bottles. They were transported to laboratory by an ice-cooled container for analysis. In the laboratory, samples in bottle were homogenized by gently inverting the bottle a couple of times. A subsample of 20 ml was then transferred to a plankton chamber and precipitated before observation. Identification and enumeration of phytoplankton taxa were then carried out under an inverted microscope at 400 × magnification (see more details in the Supplementary Materials).

Given the much higher abundance of diatom cells than dinoflagellates (typically over 10–100 folds), community composition was analyzed separately for these two key phytoplankton groups. Monthly station-level community structure was revealed at the species level with the relative abundance of the 10 most dominant (top 10) species. Community temporal (i.e. month and year) and spatial (i.e. station and WCZ) dynamics were assessed through community dissimilarity (Bray–Curtis dissimilarity, using “vegan” package in R software) and tested for significant differences using analysis of similarity (ANOSIM, “vegan” package in R) [34, 35].

### Phytoplankton size measurement and characterization

Phytoplankton body size was quantified as biovolume, with detailed method of sample treatment and measurement was in the Supplementary Methods. Size trends were analyzed at both species and community levels across spatial scales. For species-level analysis, mean biovolume was calculated for each species and plotted against lagged months (i.e. the number of months between the current sampling time and the first sampling time), with the significant trend indicated by the slope of linear regression. The 25 most abundant species (top 10 of diatoms, top 10 of dinoflagellates, and top 5 of other groups) were selected as representatives to show the long-term trends of species biovolume. Temporal trends of species biovolume were analyzed at both station and the entire region scales.

At the community level, community mean size was calculated as total biovolume divided by total cell abundance of all species for each sampling time at each station (and WCZ). Then, the community mean size was plotted against lagged months at each station (and WCZ), with the significant temporal trend indicated by the slope of linear regression.

### Calculation of Small%

According to our framework, apart from species biovolume, Small% was the other part contributing to the changes of community mean biovolume. Here, small species were defined as those located at the last quartile of all species ranked by their average biovolumes across all communities [36]. The long-term trend of proportion of small species was plotted with lagged months at each station (and WCZ) with significant trends indicated by the slope of linear regression.

### Statistical and model analyses

A raw time-series typically includes both long-term trends and seasonal variations. Since our data on species biovolume, community mean biovolume and Small% were collected through sampling at equal time intervals (i.e. monthly), strong autocorrelation may exist, supporting the use of decomposition analysis. Therefore, to reveal the underlying long-term trend, we employed Locally Weighted Regression (LOESS) to decompose the time-series data into trend, seasonal, and residual components. Given that the time granularity was monthly, the period was set to 12, indicating an annual cyclical pattern. The results of this decomposition analysis were subsequently compared with those from a direct linear regression.

Species-level responses of phytoplankton size to environmental factors were assessed using Pearson correlation coefficients (*r*) for the top 25 abundant species. A further hierarchical clustering of species based on the correlation patterns was conducted to reveal species groups with similar environmental responses (by “pheatmap” package in R) [37].

To identify the key environmental factors driving the temporal trends of phytoplankton size at both species and community levels, we compared the performances of five typical machine learning or deep learning models on the prediction of species biovolume and Small%, respectively. These models included Ridge regression (RR), Random Forest (RF), ﻿Extreme gradient boosting (XGBoost), Artificial neural network (ANN), and Support vector machine (SVM). The model with best performance was used for the downstream analyses.

To further interpret and visualize the model results, we employed the SHapley Additive exPlanations (SHAP) method, a robust game theory-based approach for quantifying feature contributions [38]. SHAP analysis operates on the principle of cooperative game theory, where each feature (here refers to environmental factor) acts as a “player” contributing to the final prediction. By evaluating all possible feature combinations, SHAP assigns comparable values that represent each predictor’s marginal contribution. Using SHAP analysis, we (i) quantified and ranked the global importance of each feature; (ii) showed the local effects (positive, negative, and intensity) of each feature in each sample, along with feature gradient; (iii) reflected the interaction effects between features, particularly relevant for phytoplankton dynamics (e.g. temperature-nutrients interactions).

To examine the partial effects of environmental drivers on species biovolume and Small%, we implemented Generalized Linear Mixed Effects Models (GLMMs). GLMMs extend traditional linear models by incorporating the variance of random predictor variables and show the “pure” effects of a certain factor on the response variable by keeping other factors constant. Here, we used GLMM to (i) test the TSR rule on phytoplankton by assessing the partial effects of temperature on species biovolume and (ii) compare the relative importance between environmental factors (e.g. temperature vs. nutrients) on regulating small%.

**Figure 2:**
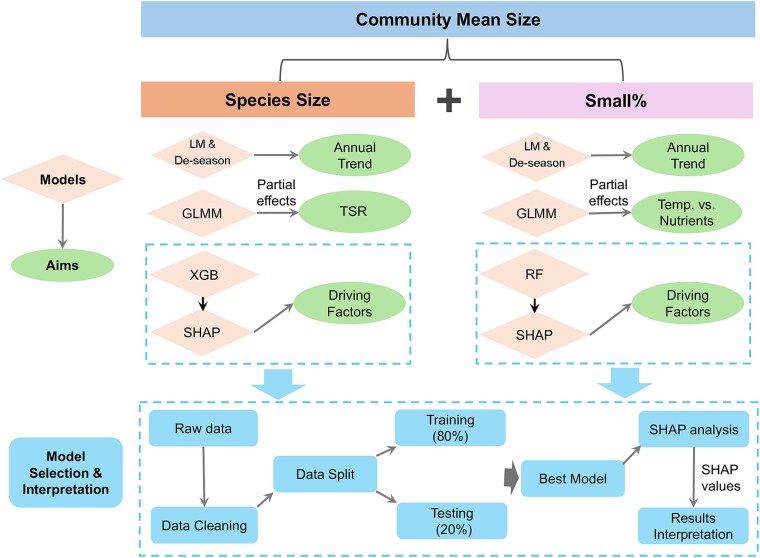
Modeling framework and ecological objectives. Machine learning approaches were selectively employed to analyze drivers of species size (XGBoost/XGB model) and Small% (Random Forest/RF model). Model selection was based on comparative performance evaluation using testing data (20% random subset), with optimal models identified through the “Model Selection and Interpretation” process (blue-colored workflow components). LM: linear model, De-season: decomposition of time-series by removing seasonality; GLMM: generalized linear mixed effects models, XGB: XGBoost, RF: random forest, Temp.: temperature, TSR: temperature-size relation.

A summary of all models, along with their ecological objectives, is presented in Fig. 2. Key steps included linear regression and time-series decomposition to quantify annual trends in size (both species and community levels) and Small%, GLMMs to disentangle the partial effects of temperature (TSR) and nutrients, and SHAP analyses based on machine (or deep) learning models to identify key driving factors.

## Results

### Long-term trends of major environmental factors

Temperature showed an increasing trend at all stations, suggesting a general pattern of warming (Fig. S1). Significant slopes (linear regression, *P* < .05) were detected at the stations near inland (e.g. TM3 to TM8 within Tolo Harbour and VM1 to VM7 within Victoria Harbour) and near the Pearl River (e.g. DM3 and DM5 within Deep Bay). The highest warming rate was recorded at station DM5, with an increase of 0.064°C per year. In contrast, no significant increase of temperature was observed at stations close to the open ocean (e.g. MM14, MM16 in Mirs Bay, and SM3 to SM19 in Southern).

The long-term trends of dissolved inorganic nitrogen (DIN), phosphate (PO_4_^3−^) and N/P ratio revealed different patterns. DIN concentrations showed regional variability (Fig. S2). Significant increases occurred in Deep Bay (DM3 and DM5), Northwestern (NM1 and NM3) and Western Buffer (WM2 and WM3) waters whereas Tolo Harbour (TM3–TM8) and Victoria Harbour (VM1–VM7) exhibited significant decreases (all *P* < .05, linear regression). No significant change of DIN was observed at stations close to the open ocean, such as MM2 to MM17 in Mirs Bay (*P* > .05).

Significant decreasing trends in PO_4_^3−^ concentrations was observed at all stations, with the sharpest decline in Tolo Harbour (e.g. TM4 with a decline rate of 1.55 μg/L per year) (Fig. S3). A widespread decline in PO₄^3−^ was confirmed by data from 2000 to 2020, which includes our phytoplankton sampling period (Fig. S4A). As a result, over the past decade (from 2010 to 2020), the Tolo Harbour stations (i.e. TM3 to TM8) and two adjacent stations (i.e. MM2 and MM17) showed the lowest concentration of PO_4_^3−^ (on average of 5.72 μg/L), compared to other regions (e.g. the highest values at Deep Bay with an average of 60.24 μg/L) (Fig. S4B).

The N/P ratio, derived from DIN divided by PO_4_^3−^, showed a consistent increasing trend at all stations (Fig. S5). The rise in N/P ratio reflected the great impacts of PO_4_^3−^ removal, as well as suggested the relative P-limitations (>16, exceeding the Redfield value) at most stations in recent years.

### Phytoplankton species composition and community dynamics

We identified and recorded the density and biovolume of 169 phytoplankton species in this study, including 91 diatom species, 60 dinoflagellate species, and 18 species from other taxonomic groups, such as cryptophytes (e.g. *Plagioselmis prolonga*), dictyochophytes (e.g. *Dictyocha fibula*), and cyanobacteria (e.g. *Trichodesmium erythraeum*).

Diatoms dominated the phytoplankton community in absolute abundance, contributing an average of 39.5% (SD = 28.38%) of total cell counts, whereas dinoflagellates represented only 4.01% (SD = 5.80%)—approximately one order of magnitude lower (Fig. S6). The high standard deviations in both relative and absolute abundances (evident from the intensive peaks in Fig. S6) highlight strong spatiotemporal variability within phytoplankton communities.

Given the marked disparity in abundance between diatoms and dinoflagellates, we showed their community structure separately. Represented by the relative abundance of top 10 most abundant species, their communities displayed apparent spatial and temporal dynamics (Figs S7 and S8).

For the community structure of the whole phytoplankton, annual difference (ANOSIM *R* = 0.19, *P* < .001) was higher than seasonal difference (ANOSIM *R* = 0.11, *P* < .001) and spatial difference (ANOSIM *R* = 0.12 and 0.15 for WCZ and station, respectively, both *P* < .001), reflecting a long-term shift in community compositions (Fig. S9).

### Phytoplankton miniaturization at the species and community levels

**Figure 3:**
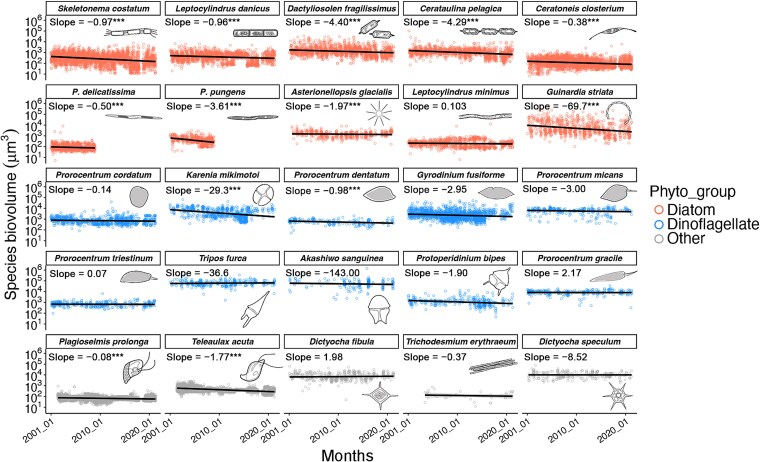
Phytoplankton miniaturization at the species level. Size dynamics (biovolume in μm^3^) are shown for the 25 most abundant species: top 10 diatoms (red circles), top 10 dinoflagellates (blue circles) and top 5 other phytoplankton (gray circles; including cryptophytes, dictyochophytes, and cyanobacteria). Species miniaturization was quantified through linear regression of time (lagged months) versus species biovolume (slope < 0, *P* < .05) with samples from the entire region. Trendline significance: ^*^: *P* < .05, ^**^: *P* < .01, ^***^: *P* < .001. Schematic illustrations (right margin) show representative morphology (not to scale).

At the species level, a pervasive miniaturization trend was observed across dominant phytoplankton species (Fig. 3). Among the 25 most abundant species (10 diatoms, 10 dinoflagellates, and five other groups), nine diatom species showed significantly decreasing trends in biovolume over time whereas only 2 dinoflagellate species (*Karenia mikimotoi* and *Prorocentrum dentatum*) and two species from other phytoplankton groups (*P. prolonga* and *Teleaulax acuta*, both are cryptophytes) exhibited similar reductions (linear regression: slope < 0, *P* < .001). Multiple testing of *P* values using Benjamini-Hochberg (BH) confirmed the results, with *q* values <0.05 for all the 13 species (except *Asterionellopsis glacialis* with *q* values = 0.14) with significantly decreasing size trends. None of the analyzed species displayed significant biovolume increases. This result was supported by the time-series decomposition analysis, showing the significant decreasing trend of these species after removing seasonality effects (Fig. S10). At a finer spatial scale, station-specific species miniaturization was widespread across all sampling sites (Fig. S11A). Diatoms demonstrated the strongest response, with 93% of station-specific species (141 of 151) showing significant size reduction, compared to 58% of dinoflagellates (72 of 124) and 86% of other groups (74 of 86) (Fig. S11B). Consistent with this pattern, annual relative changes in biovolume over the 20-year period showed diatoms undergoing the most substantial size decline (Fig. S12).

**Figure 4:**
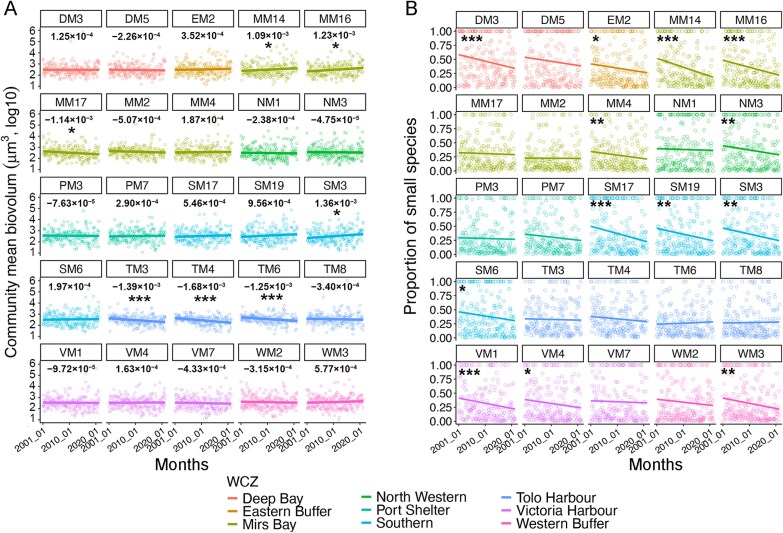
Phytoplankton community-level miniaturization patterns. **(A)** Shows the temporal trends in community mean biovolume across the 25 monitoring stations, with negative trends indicating community miniaturization. Slope of the trends are shown below the station label (μm^3^/year). **(B)** Shows the temporal trends in Small%, the secondary driver of community miniaturization. Stations are colored by water control zones (WCZs). Significantly trends are marked with codes (^*^: *P* < .05, ^**^: *P* < .01, ^***^: *P* < .001, by linear regression).

Phytoplankton miniaturization at the community level was observed only at the stations (i.e. TM3, TM4, and TM6) within the semi-closed bay of Tolo Harbour and a nearby station MM17 (linear regression: slope < 0, *P* < .05) (Fig. 4A), supported by the time-series decomposition analysis (except VM7, which also showed significant miniaturization, Fig. S13). In contrast, stations with greater oceanic influence (MM14, MM16, SM3) exhibited significant increases of community mean biovolume (slope > 0 and *P* < .05). This spatial pattern was consistent at the WCZ scale, where only Tolo Harbour showed significant community miniaturization (slope < 0, *P* < .001) (Fig. S14).

### Widespread declines in Small%

No station showed a significant increasing trend in Small% over the years (Fig. 4B). Instead, more than half of stations (13 of 25) exhibited significant Small% decreases (linear regression: slope < 0, *P* < .05), indicating a shift toward larger species. Stations in or near Tolo Harbour region (TM3, TM4, TM6, TM8, MM17, and MM2) showed no significant changes of Small% (*P* > .05), reflected by both direct linear regression (Fig. 4B) and time-series decomposition analysis (Fig. S15). This pattern was consistent at the WCZ scale, with all zones except Tolo Harbour showing significant Small% declines (Fig. S16).

The widespread decreases in Small% could be attributed to the reduced relative abundance of species from both diatoms and dinoflagellates (Fig. S17A). For instance, *Skeletonema costatum*, the most abundant diatom species*,* showed an annual decrease in relative abundance of 0.25% per year. Significant declines were also detected in three abundant species belong to dinoflagellate genus *Prorocentrum*, including the dominant *P. cordatum* (linear regression: slope < 0, *P* < .05). In contrast, we observed more increasing than decreasing trends among larger species, particularly within the dinoflagellate genus *Tripos* and for *Akashiwo sanguinea* (linear regression: slope > 0, *P* < .05) (Fig. S17B).

### Environmental factors driving phytoplankton miniaturization

**Figure 5:**
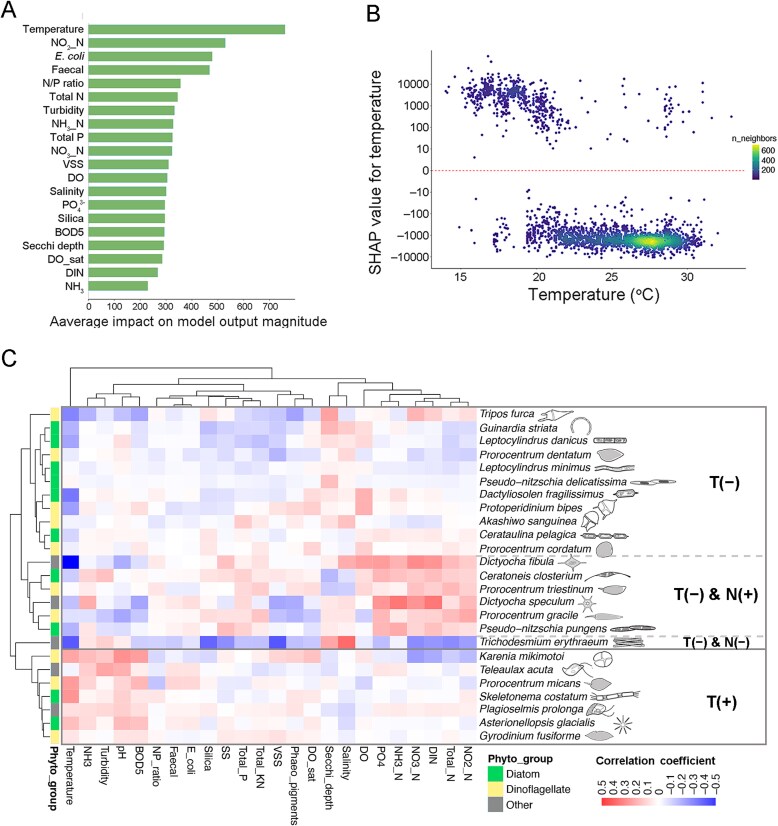
Effects of environmental factors on species biovolume. **(A)** The relative importance of each environmental factor on the species biovolume was assessed and ranked using SHAP analysis. A larger average impact on model output magnitude means a greater contribution to species biovolume variation. **(B)** SHAP values illustrate the influence of temperature on species biovolume across temperature gradients, where positive (or negative) SHAP values denote positive (or negative) effects. **(C)** The top 25 abundant species were clustered based on correlations between their temporal biovolume variations and environmental factors (*n =* 24) (by Pearson correlation coefficient, red = positive correlation, blue = negative correlation). Phytoplankton groups are color-coded on the left (green: diatom; yellow: dinoflagellate; grey: others). These species are hierarchically classified into four categories: (i) negative to temperature (T$-$); (ii) negative to temperature and positive to nutrients (T$-$ & N$+$); (iii) negative to temperature and negative to nutrients (T$-$ & N$-$); (iv) positive to temperature (T$+$).

At the species level, the summary plot of the feature importance revealed that temperature was the most important factor regulating the species biovolume (by XGBoost model which performed the best in this step, Table S2). This was evidenced by its substantially higher mean absolute SHAP value compared to other environmental factors (Fig. 5A). Temperature showed negative effects on species biovolume in most of the samples especially at high temperatures (Fig. 5B).

In addition to the negative effects from temperature, positive relations were also found between biovolume and temperature for several species, as well as the regulations from nutrients. By clustering of the top 25 abundant species based on the responses of their biovolumes (Pearson’s *r*) to different environmental factors, these species could be classified into four groups: (i) mainly negative to temperature (*n =* 11); (ii) negative to temperature and positive to nutrients (*n =* 6); (iii) negative to temperature and negative to nutrients (*n =* 1, i.e. *T. erythraeum*); (iv) mainly positive to temperature (*n =* 7) (Fig. 5C). The absence of consistent phylogenetic clustering (at either phytoplankton group or genus level) suggests diverse long-term adaptation strategies among species.

**Figure 6:**
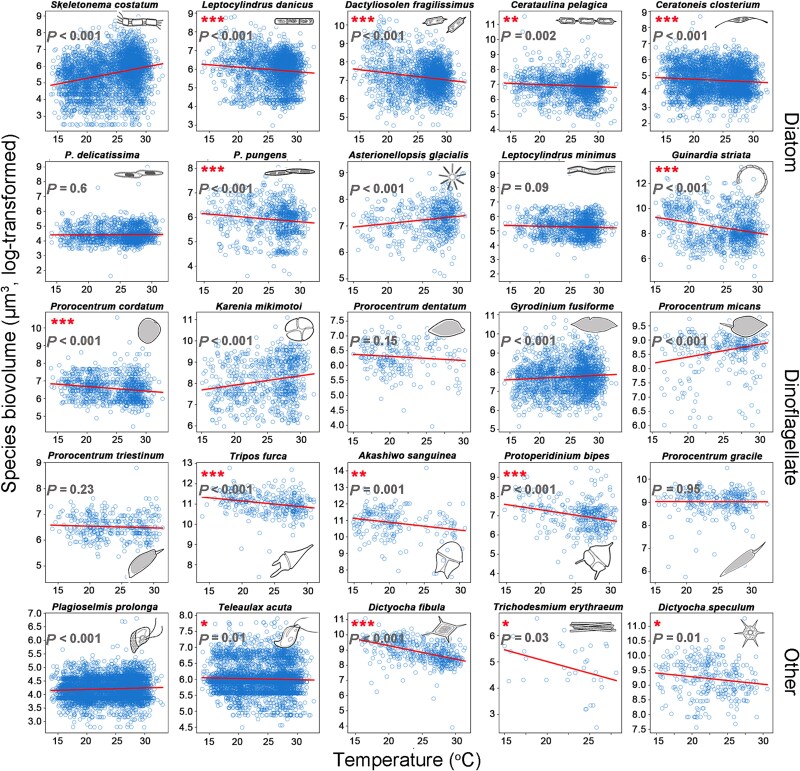
Temperature-biovolume relationships for the top 25 abundant species. Red solid lines represent the fitted values predicted for each species using GLMM (with all other environmental factors held constant). Statistical significance is indicated at the top-left corner: ^*^  *P* < .05; ^**^  *P* < .01; ^***^  *P* < .001 (significantly negative trends, matching TSR, are marked with red asterisks). Note: y-axis scales vary across species due to differences in body size ranges. *P*.: *Pseudo-nitzschia.*

TSR was tested for the top 25 abundant species using partial effects analysis (by GLMM, holding all other factors constant) (Fig. 6). The results revealed significant negative effects of temperature on the biovolume of 14 species (linear regression: slope < 0, *P* < .05), supporting TSR. These included six diatoms (*Leptocylindrus danicus*, *Dactyliosolen fragilissimus*, *Cerataulina pelagica*, *Ceratoneis Closterium*, *Pseudo-nitzschia pungens*, and *Guinardia striata*), four dinoflagellates (*P. cordatum*, *T. furca*, *A. sanguinea,* and *Protoperidinium bipes*)*,* and four others (*T. acuta*, *D. fibula*, *T. erythraeum*, and *D. speculum*). Conversely, temperature had positive effects on six species (2 diatoms including *S. costatum* and *A. glacialis*, three dinoflagellates including *K. mikimotoi*, *Gyrodinium fusiforme*, and *P. micans* and 1 other species *P. prolonga,* slope > 0 and *P* < .05), contradicting TSR.

**Figure 7:**
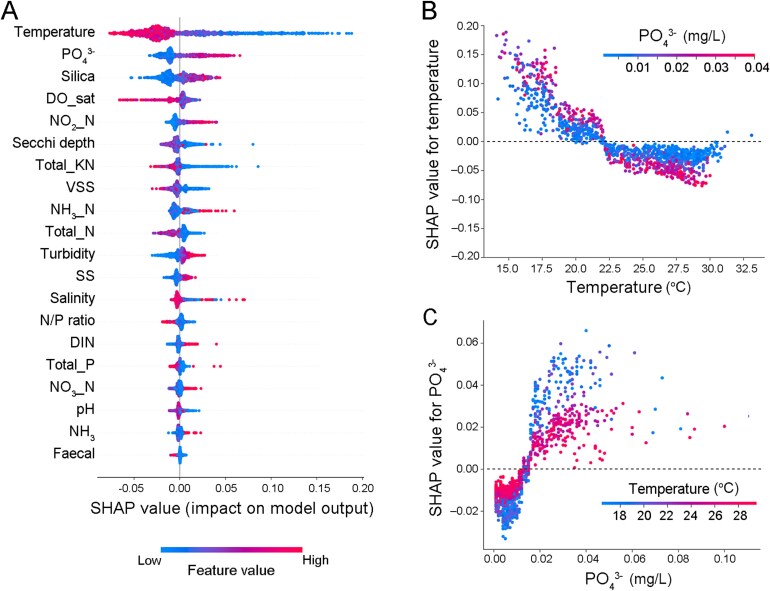
Effects of environmental factors on Small%. **(A)** SHAP summary plot of top 20 environmental factors ranked by their mean absolute SHAP values, where higher rank indicates greater impact on the model prediction. SHAP values represent effect directions (positive/negative) and magnitudes (absolute values), with individual samples shown as dots colored by feature values (blue: low; red: high). **(B, C)** Interactive effects between temperature (°C) and phosphate (PO_4_^3−^, mg/l) on Small%. Positive (or negative) SHAP values indicate positive (or negative) effects on Small% and larger absolute values of SHAP mean higher impacts.

Temperature was the most important factor influencing Small%, with generally stronger negative effects than positive effects, as determined by the Random Forest method (the best-performing model in this step, see Table S2) and SHAP analysis (Fig. 7A). PO_4_^3−^ ranked second in importance, exhibiting particularly strong positive effects at elevated concentrations. Interactive effect analysis between temperature and PO_4_^3−^ revealed enhanced temperature impacts (both positive and negative) on Small% under high PO_4_^3−^ concentrations (Fig. 7B), indicating that nutrient levels may influence the temperature sensitivity of phytoplankton community. The effects of temperature transitioned from positive to negative when temperature exceeded ~22°C, though these high-temperature negative effects remained minimal (approaching zero). PO_4_^3−^ had negative effects on Small% at low concentrations and showed strong positive effects when concentrations exceeded 0.02 mg/l. Furthermore, its effects on Small% were relatively higher at lower temperature conditions (Fig. 7C).

Partial effects analysis by GLMM showed that PO_4_^3−^ had significantly positive effects on Small% at 15 stations whereas significant influences from temperature were only found at 12 stations, suggesting the key role of PO_4_^3−^ in controlling the phytoplankton community turnover. The semi-closed Tolo Harbor region (TM3 to TM8) and its nearby stations (MM17 and MM2) showed no significant response to either environmental factor (both *P* > .05, partial effects) (Fig. S18 and S19).

## Discussion

### Long-term species miniaturization suggesting diverse evolutionary adaptation

Our work provides a comprehensive analysis of phytoplankton species size variations using a long-term dataset spanning 20 years, offering insights rarely available in previous research [39, 40]. The monthly sampling design with balanced temporal coverage enabled us to characterize annual phytoplankton size trends while effectively minimizing seasonal biases that have constrained most previous studies of phytoplankton temporal dynamics. Unlike laboratory-based experiments that are inherently limited in species representation, our field observations captured size trends across the entire spectrum of microscopically identifiable species within natural phytoplankton communities. This provided an unprecedented opportunity to analyze the interspecific variations within and among phytoplankton groups, facilitating robust community-level assessments of size changes.

By quantifying the annual trend in species biovolume with linear regression, our dataset revealed a clear pattern of phytoplankton miniaturization at the species level. This was evidenced by the significantly reduced size of over half of the abundant species at the regional scale and the negative average size trend across most stations at the local scale. The annual size trends varied even among species within the same genus, such as *Leptocylindrus* in diatoms and *Prorocentrum* in dinoflagellates, suggesting that phytoplankton miniaturization should be examined at the species or finer taxonomic levels to accurately capture the long-term adaptations. The high variability in size plasticity among phytoplankton species further underscores the importance of interspecific (and intraspecific) diversity to maintain ecosystem functions under climate changes [41].

Our results showed that species miniaturization was more pronounced in diatoms than in dinoflagellates (Fig. 3, S11B). Most of diatom species are r-strategists with high sensitivity to environmental changes, facilitating more rapid phenotypic adaptations. Reduced cell size can serve as a strategy to enhance homeostasis and nutrient acquisition for marine algae [42]. Temporal changes in diatom cell size, typically measured as biovolume or biomass, have been frequently documented in the micro- or mesocosm experiments to show the effects from manipulated gradients of environmental stressors (e.g. temperature, light, nutrients, and grazing pressure) [8, 43], but receive little attentions in long-term field survey. Given the predominance of diatoms in coastal waters, their size reduction may have profound ecological implications. First, smaller diatom cells would contain less carbon (per biovolume) and exhibit lower sinking velocities (as predicted by Stoke’s Law), favoring carbon retention in the water column over sedimentation [44–46]. Second, shifts in prey size spectrum could significantly alter predator–prey dynamics (e.g. grazing rate and feeding relationships, particularly for filter-feeding zooplankton) in marine food webs.

Cell sizes of both dinoflagellates and diatoms are largely regulated by temperature, nutrient levels (e.g. N- and P-limitation) and their interaction effects [8, 21]. Many dinoflagellates are mixotrophic, primarily relying on photosynthesis under nutrient-replete conditions but switching to facultative heterotrophic under nutrient limitation. This metabolic flexibility may allow dinoflagellates to respond more resiliently to environmental stress, resulting in less pronounced size changes compared to diatoms, particularly in terms of nutrient acquisition. In our study, all the top 10 abundant dinoflagellate species (except *P. dentatum*) have been documented as (potential) mixotrophic [47, 48], possibly explaining the weaker species-level miniaturization in dinoflagellates relative to diatoms.

### Drivers of phytoplankton size: which ecological rule to follow?

Using partial effects analysis (GLMM), we found that TSR was significant in more than half of the abundant phytoplankton species (Fig. 6). Long-term evidence of TSR in phytoplankton species has been reported in the fossil records of diatoms, showing reduced frustule sizes under warmer conditions, as well as in sedimentary archives of dinoflagellate cysts [49, 50]. TSR in marine phytoplankton were tested by previous studies using laboratory incubation experiments and large-scale field surveys [2, 42, 51]. Particularly, Atkinson *et al.* [42] observed an inverse relationship between cell volume and temperature in multiple protistan species, reporting a ~2.5% reduction in cell volume per 1°C increase from a baseline of 15°C. They attributed this size reduction to phenotypic plasticity rather than evolutionary adaptation due to the short experimental period (days to weeks). In contrast, our study quantified TSR over a 20-year period, capturing evolutionary adaptations under climate change. This distinction likely explains why Atkinson *et al.* [42] found consistent size reduction rates across species whereas our results revealed high interspecific variability in size trends, reflecting diverse evolutionary adaptation in coastal phytoplankton species. Furthermore, temperature-driven shifts in the unique life cycles of phytoplankton species may also contribute to the observed changes in body size. For instance, diatoms undergo a programmed size reduction until reaching a species-specific threshold that triggers sexual reproduction, and cryptophytes exhibit significant cell size variation between life stages, with diploid cells being substantially larger than haploid ones. Since temperature can directly influence the duration of these life cycle stages, it is a plausible factor in the overall size distribution [43, 52].

Using GLMM analysis, we evaluated the partial effects of temperature on biovolume by controlling for all other environmental factors (potentially collinear), providing a robust quantification of TSR—an approach rarely employed in previous studies. Our results showed that, for the top abundant phytoplankton species, six out of 10 diatom species and four out of 10 dinoflagellates species followed TSR (Fig. 6). This supports the opinion that the negative relationship between size and temperature, including TSR, is not universal for all organisms. Exceptions to TSR were reported in phytoplankton species [53, 54], which could be explained by oxygen shortage, grazing pressure, and their unique life cycles with seasonal constrains [55]. For instance, certain diatom species, such as *S. costatum* (the most abundant species in our data), may exhibit size increases at high temperatures due to vegetative enlargement, increasing viability without triggering sexual reproduction [56]. Additionally, while nutrient limitation typically reduces phytoplankton body size, P-limitation has often been reported to cause cell enlargement in dinoflagellates [57–59], potentially regulated by cell cycle arrest via specific gene expression [60]. Therefore, the widespread P-limitation in Hong Kong waters, evidenced by the annual declining phosphate concentrations and rising N:P ratios (Figs S3–S5), may explain the weaker miniaturization and TSR signal in dinoflagellates compared to diatoms in our results.

Unlike TSR, which describes the body size change of species, Bergmann’s rule in our study was interpreted in a broad sense, referring to the decrease of body size at the community level under warming. We tested Bergmann’s rule by quantifying the replacement of large-sized species by small species, which was analyzed for each station to show the long-term trend of species exchange. Unexpectedly, our results contradicted Bergmann’s rule, showing a significant decline of Small% at most stations despite regional warming. This was corroborated by the GLMM results, which indicated negative or nonsignificant partial effects of temperature on Small% (Fig. S18).

The first reason for the declined Small% in our study could be the limitation of microscope observation. Pico-sized (0.2–2 μm) phytoplankton, which typically dominated in warmer waters [5], were not included in our data. In other words, the “small species” in our study referred to nano-sized phytoplankton, which were around 5 μm in equivalent spherical diameter ESD (Fig. S17). Nano- or micro-sized phytoplankton, usually have higher concentrations of chlorophyll *a* than picophytoplankton in productive coastal waters underscoring their ecological relevance [29, 61–63]. Previous studies have reported the decrease of nanophytoplankton relative abundance at high temperatures [25, 64], which aligns with our findings. Specifically, *S. costatum*, the most abundant diatom species frequently forming HAB in Hong Kong coastal waters, was defined as small species here and showed a significant annual decrease in relative abundance, contributing greatly to the reduced Small% in our study. One possible reason could be that *S. costatum* could only use much fewer types of phosphate than other phytoplankton species [65], and may encounter photoinhibition under P-limitation, causing community shift to other larger-sized dominant diatom species [66].

The second reason could be the regulation of PO_4_^3−^ concentration on Small%, as indicated by its second-highest rank of importance in the SHAP analysis (Fig. 7). The positive correlation between PO_4_^3−^ levels and relative abundance of nanophytoplankton has been found in both field survey [67] and nutrient amendment experiments [68], highlighting the key roles of PO_4_^3−^ in controlling coastal phytoplankton community structure. Similarly, our findings showed that Small% (nano-sized species here) decreased annually under the ubiquitously decline of phosphate concentration (Fig. S3). This trend opposes our species-shift hypothesis but helps explain the lack of community-level miniaturization despite widespread species-level size reductions. Furthermore, we showed that temperature had weak impacts on Small% when PO_4_^3−^concentration was low, suggesting that nutrient levels may influence the temperature sensitivity of phytoplankton community. This nutrient-dependent temperature effects on phytoplankton community size structure have been scarcely reported but can be reasonable because the thermal responses (e.g. growth rate) of phytoplankton, especially diatoms, are largely influenced by nutrient availability [69]. These findings highlight the importance of considering the combined effects of environmental factors when predicting the responses of marine ecosystem to global warming.

### Spatial differences in miniaturization: implications for environmental protection

Community-level miniaturization was not observed at most of the stations, negating our first hypothesis. While species miniaturization of phytoplankton, mainly driven by temperature, may widely occur under global warming, our results suggest that historical nutrient reduction could mitigate community-level miniaturization by decreasing the proportion of small species in coastal waters. This would have positive ecological significance on marine food web as miniaturization of phytoplankton could lead to a decreased efficiency of the biological carbon pump [2].

However, phytoplankton miniaturization was significant in the semi-closed bay (i.e. Tolo Harbour) at both species and community levels (Fig. 4A, S11A). To reduce pollution loadings, the Tolo Harbour Action Plan (THAP) was implemented since 1987, followed by Tolo Harbour Effluent Export Scheme (THEES) since 1995 (EPD, Hong Kong). Due to these efforts on nutrient control in Tolo Harbour, though the water quality has improved markedly, the concentration of PO_4_^3−^ (0.06 μM, on average) has reduced, being the lowest among all WCZs in recent years (Fig. S4B) and can be regarded as P-limitation according to previous studies. (e.g. the threshold of 0.2 μM reported in [70, 71]). Despite this low level of PO_4_^3−^, Small% didn’t change significantly in Tolo Harbour. One indirect reason could be the interactive effects between temperature and phosphate, where low PO_4_^3−^ level diminished the phytoplankton community’s response (in terms of Small%) under high temperature (Fig. 7B), weakening the relationship stated in Bergmann’s rule. This is supported by the findings in both pelagic water of Atlantic Ocean [72] and freshwater from a temperate lake [73], showing the nutrient-dependent temperature effects on the size structure of phytoplankton communities.

Another reason for the negligible changes in Small% in Tolo Harbour could be found in the GLMM results where the pure effects of PO_4_^3−^ on Small% were not significant at the stations within and near this region (Fig. S19). Given that PO_4_^3−^ usually acts as a major role in controlling the spatiotemporal dynamics of phytoplankton community structure in the coastal waters of China [29, 68], we argue that this insignificant relation could be explained by the unique geographic character of Tolo Harbour as a semi-closed bay with limited water exchange rate leading to low community turnover [74–76]. This is supported by our finding that Tolo Harbour had a high residence time of water which negatively regulated the decline rate of Small% (Fig. S20). Therefore, we suggest that though nutrient reduction could act efficiently in countering community-level phytoplankton miniaturization, it is essential to establish a balanced and appropriate target for nutrient levels. Additionally, special attention and tailored actions should be taken in semi-closed or closed waters where the community processes could be different.

## Conclusion

We quantified and revealed the long-term size changes in coastal phytoplankton at both species and community levels. At the species level, we observed widespread phytoplankton miniaturization, particularly in diatoms compared to dinoflagellates, which was primarily driven by the increasing temperature, supporting the TSR. It suggests a potential shift in carbon sequestration dynamics, favoring the water column over sediment storage, and indicates alterations in microbial loop structure such as prey–predator interactions. Besides, the different patterns of body size change among species, along with distinct major regulating factors, suggest the diverse evolutionary adaptation mechanisms between and within phytoplankton groups, even at the genus level. At the community level, however, no significant miniaturization was observed at most stations, which was contributed by decreased Small% (especially *S. costatum*). Using machine learning model combined with explainable SHAP analyses, we identified the key driving factors of long-term phytoplankton miniaturization and demonstrated their effects from both local and regional scales. We showed that the declined PO_4_^3−^ concentration not only directly reduced Small% but also weakened the warming effects on phytoplankton community size structure. Therefore, our results highlight that nutrient reduction (e.g. achieved through anthropogenic environmental management) is critical for mitigating phytoplankton miniaturization at the community level, with positive implications for HAB control and biological pump efficiency. We also addressed the different process in a semi-closed bay where community miniaturization was significant, suggesting the demand of area-specific actions.

## Supplementary Material

Supplementary_materials-revised-v6_wraf257

## Data Availability

All datasets and codes used in the above methods were deposited and available in the GitHub website: https://github.com/xzhimenghkust/Phytoplankton-miniaturization-2025
