# Supplementary material for: Decadal scale phytoplankton species miniaturization in subtropical coastal waters
Source: ISME J. 2025 Nov 20;19(1):wraf257. doi: 10.1093/ismejo/wraf257 (PMC12676728; doi:10.1093/ismejo/wraf257)
Supplement: Supplementary_materials-revised-v6_wraf257 [file supplementary_materials-revised-v6_wraf257.docx]

Supplementary Materials for

**Decadal scale phytoplankton species miniaturization in subtropical coastal waters**

**Zhimeng Xu *et al*.**

*Corresponding author Email: [liuhb@ust.hk](mailto:liuhb@ust.hk)

**This file includes:**

1. **Supplementary Methods**
2. **Supplementary Figures (Fig. S1 – S20)**
3. **Supplementary Tables (Table S1 – S2)**
4. **References (only for Supplementary Materials)**

**I Supplementary Methods**

**Phytoplankton identification, size measurement and community construction**

Prior to analysis, samples were gently homogenized several times to resuspend the cells. A 20 ml aliquot of each sample was then transferred to a Utermöhl chamber (Hydro-Bios, Germany) and allowed to settle for at least 24 hours. Following sedimentation, phytoplankton cells were enumerated using an inverted microscope (Olympus IX51) following the Utermöhl method ^1^. Taxonomic identification was performed to the lowest taxonomic level (species or genus) according to Isamu (1991), Tomas (1997), Yang et al. (2014) and Law & Lee (2013) ^2-5^. Notably, an increased subsample volume of 50 - 100 ml was analyzed to ensure data reliability when phytoplankton counts in the 20 ml subsample fell below 100 cells.

Biovolume of phytoplankton cells was calculated based on Hillebrand et al (1999) ^6^ with identification results. In brief, the length of different part of cells were measured according to the specific geometric shapes of the identified species. Biovolume were then computed using the mathematical formulas for these geometric shapes. The mean biovolume for each species was determined from measurements of at least 10 cells randomly selected individual cells. Total species biovolume was subsequently calculated by multiplying the average biovolume by the total abundance of each species.

Phytoplankton community was constructed with the abundance of each species for each sampling time (monthly from 2000 to 2020) at each station. Taxonomic nomenclature of species was standardized throughout the dataset. For instance, the names of two diatom species “*Pseudo-nitzschia delicatissima*” and “*Pseudo-nitzschia pungens*” were recorded in the first 10 years (2000-2010) while only “*Pseudo-nitzschia* spp.” was used later (2010-2020). Therefore, we changed them to “*Pseudo-nitzschia* spp” across all samples. Notably, a substantial fraction of small-flagellated phytoplankton could not be identified to species level; these were categorized as “small flagellates” and retained in analyses to maintain the complete size spectrum of the community.

**II Supplementary figures (N = 20)**

**
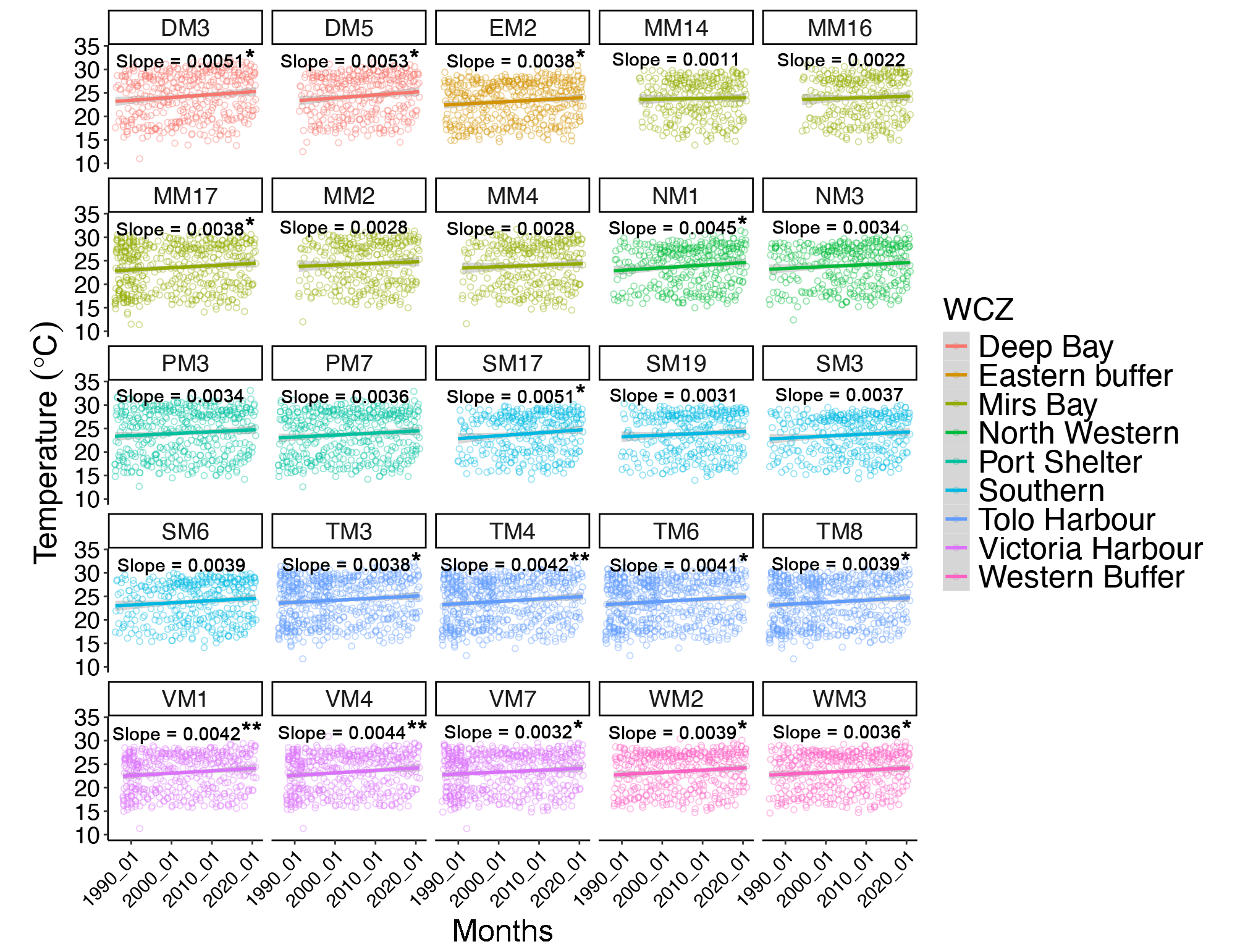
**

**Figure S1 | Long-term trends in temperature.** Monthly temperature measurements collected from 1986 to 2020 are displayed, with points color-coded by water control zone (WCZ). Trendlines (solid lines) with 95% confidence intervals (colored shading) were derived from linear regression, with slopes indicating the rate of change (°C per year). Significance code: *: *P* < 0.05, **: *P* < 0.01, ***: *P* < 0.001.

**
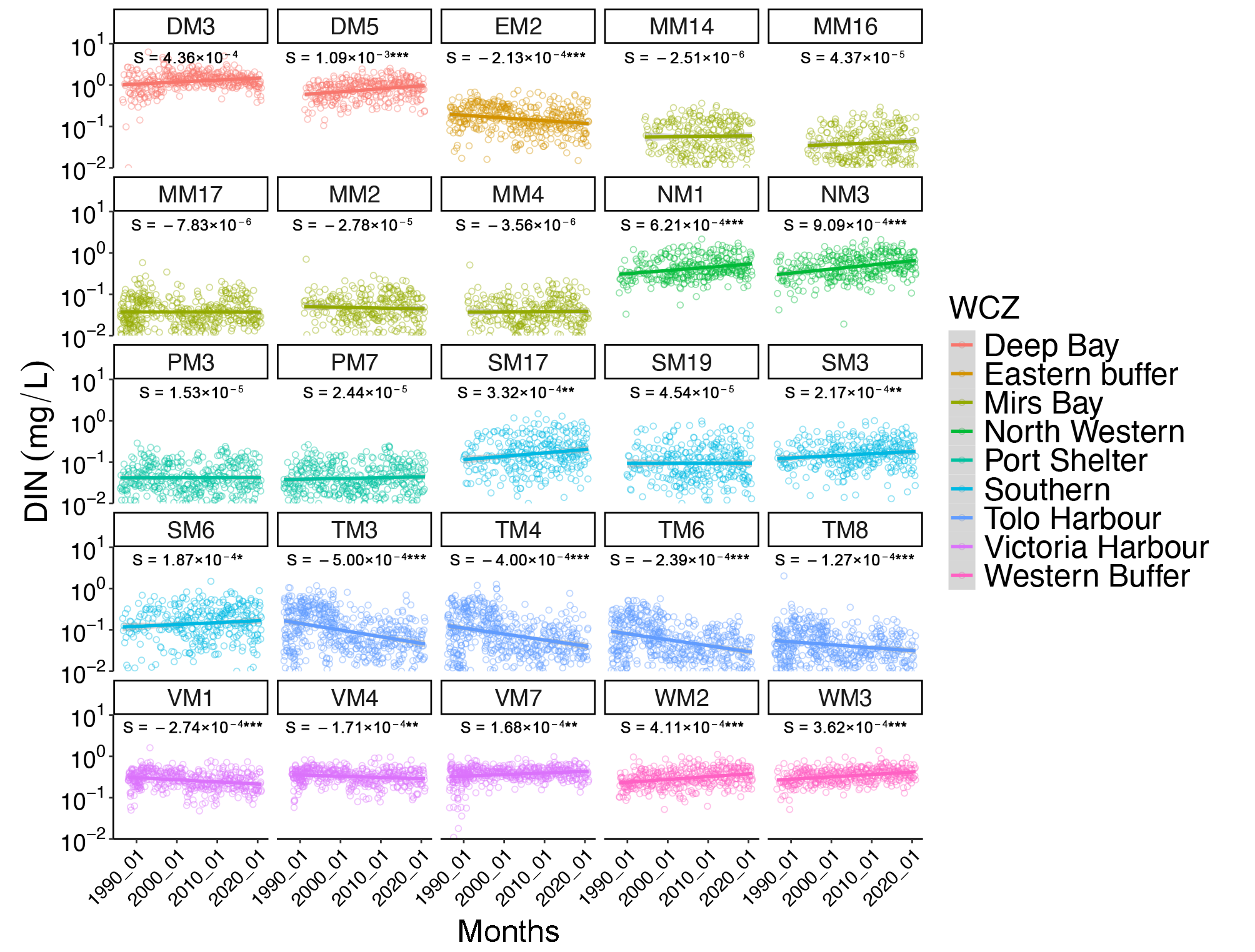
**

**Figure S2 | Long-term trends in dissolved inorganic nitrogen (DIN).** Monthly measurements of DIN (sum of NO₂⁻, NO₃⁻, and NH₄⁺, in unit of mg L⁻¹) from 1986 to 2020 are plotted, with colors indicating different water control zones (WCZs). Trendlines (solid lines) with 95% confidence intervals (colored shading) were derived from linear regression, with slopes (S) representing the rate of change (mg L⁻¹ yr⁻¹). Significance code: *: *P* < 0.05, **: *P* < 0.01, ***: *P* < 0.001.


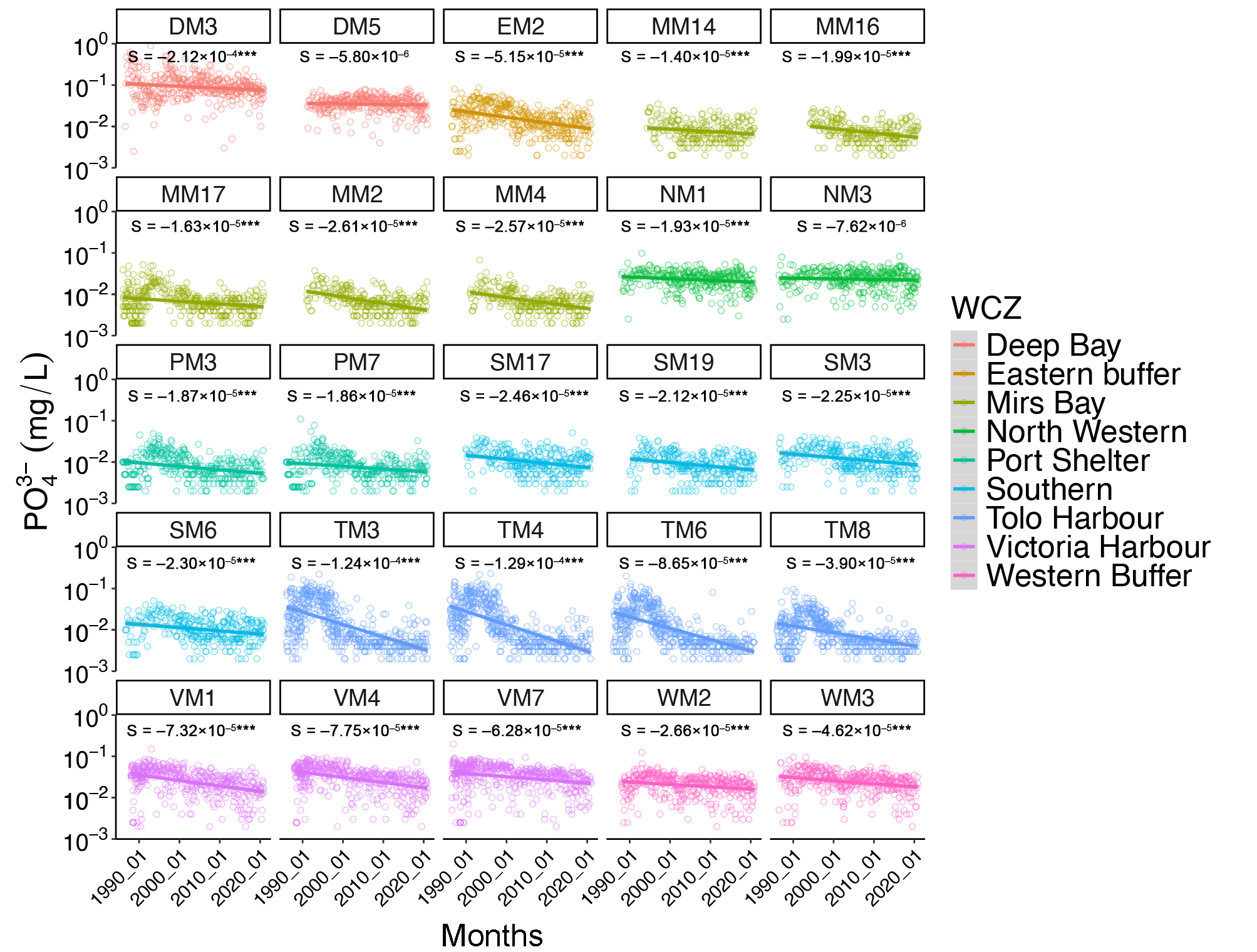


**Figure S3 | Long-term trends in phosphate concentration (PO_4_^3-^).** Monthly measurements of PO_4_^3-^ concentration (mg L⁻¹) from 1986 to 2020 are displayed, with colors distinguishing different WCZs. Solid trendlines with 95% confidence intervals (colored shading) were generated through linear regression, with slope values (S) indicating the rate of change (mg L⁻¹ yr⁻¹). Significance code: *: *P* < 0.05, **: *P* < 0.01, ***: *P* < 0.001.

**
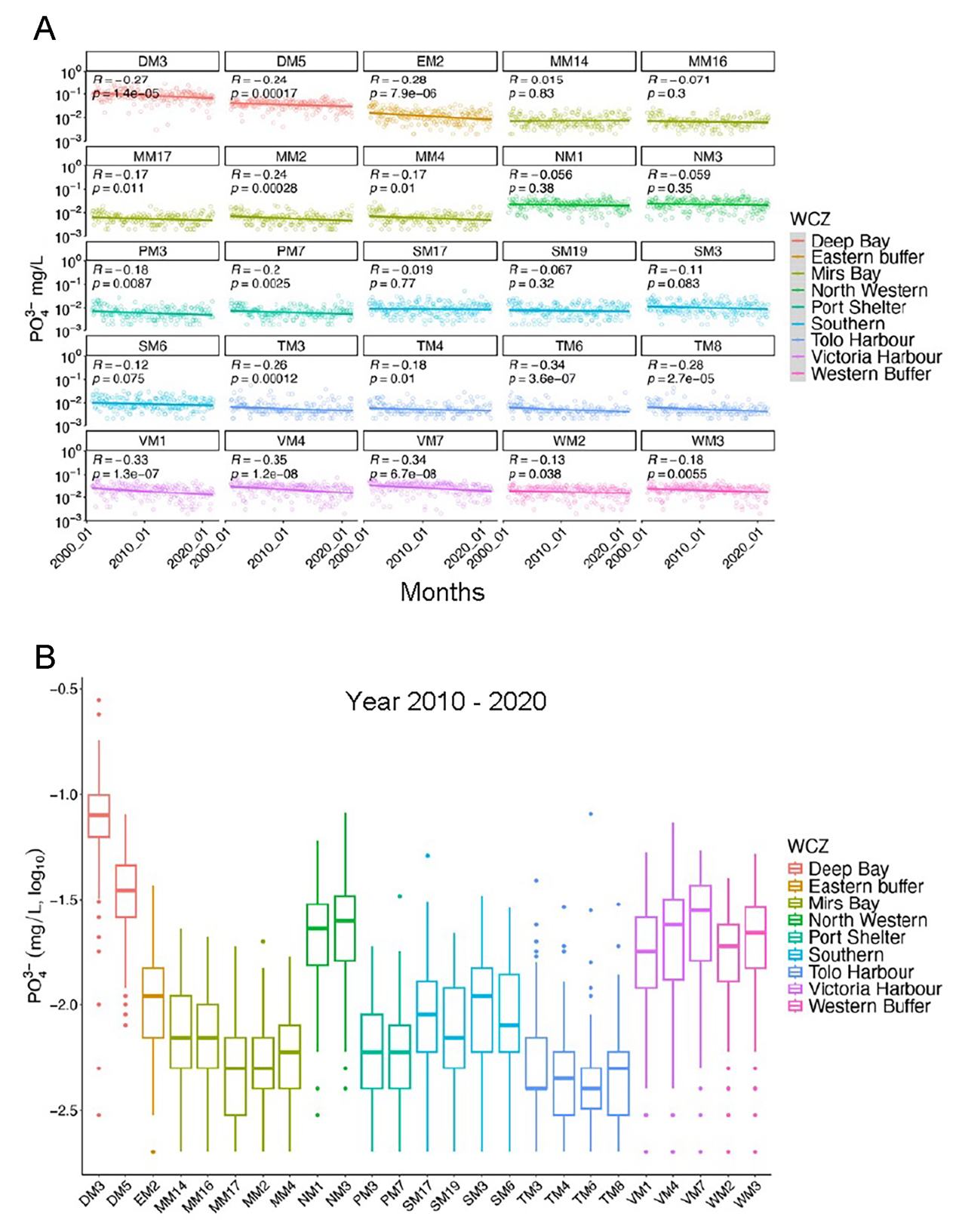
**

**Figure S4 | PO_4_^3-^ concentration among stations from 2000 to 2020 (A) and in recent 10 years (B).** Data were extracted from Fig. S3. Linear regression is used in panel **(A)**. Panel **(B)** shows the relatively low values of phosphate at stations of Tolo Harbour (in blue) during the recent decade (from 2010 to 2020). Note that concentrations of PO_4_^3-^ are log_10_ transformed.


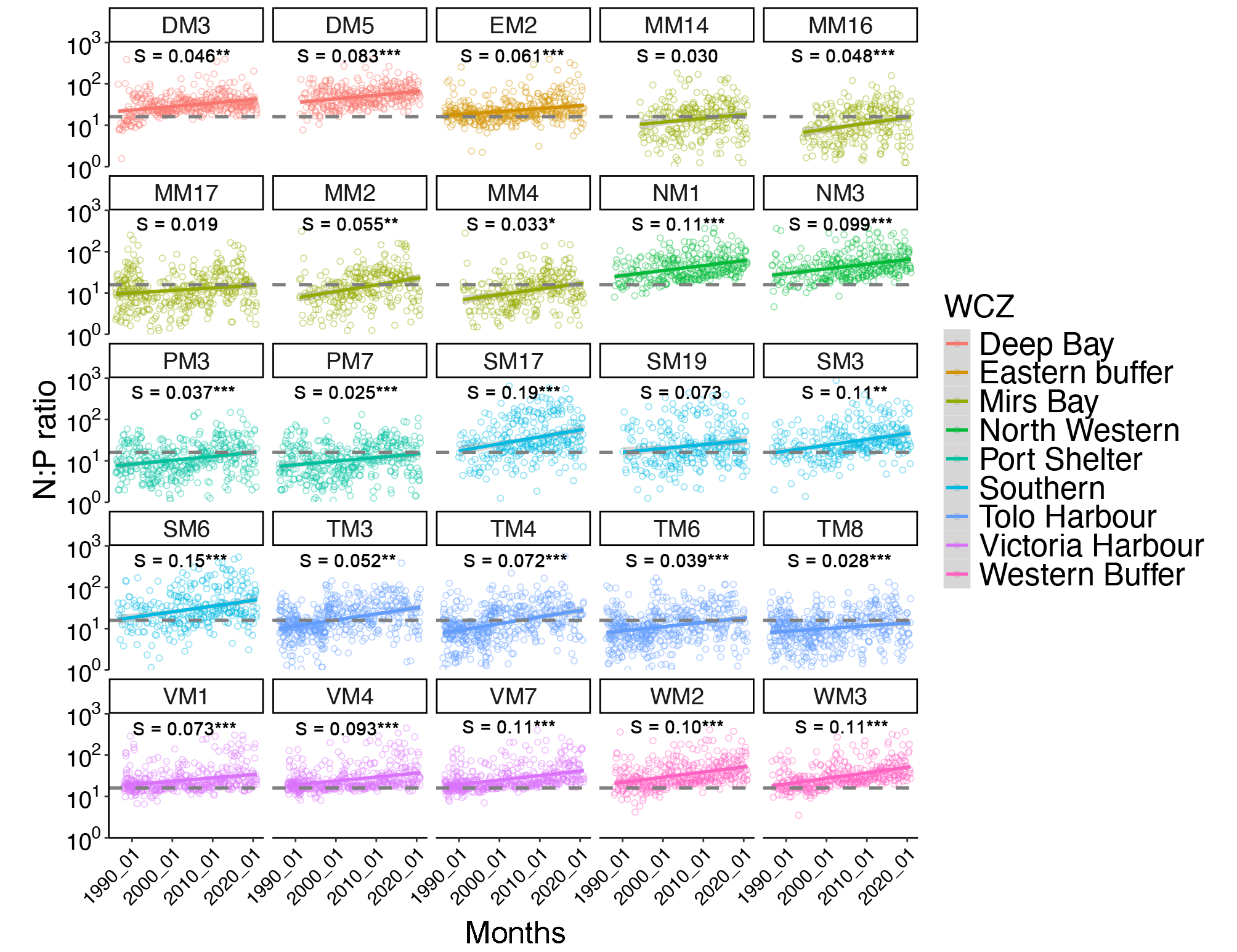


**Figure S5 | Long-term trends in N:P ratio.** N here presents DIN while P refers to PO_4_^3-^. Data are plotted with colors representing different WCZs. Annual trendlines of phosphate of N:P values are shown with slopes (S) and statistical significance from linear regression. (Significance code: *: *P* < 0.05, **: *P* < 0.01, ***: *P* < 0.001). The Redfield ratio (N:P = 16) is indicated by a horizontal grey dashed line for reference.


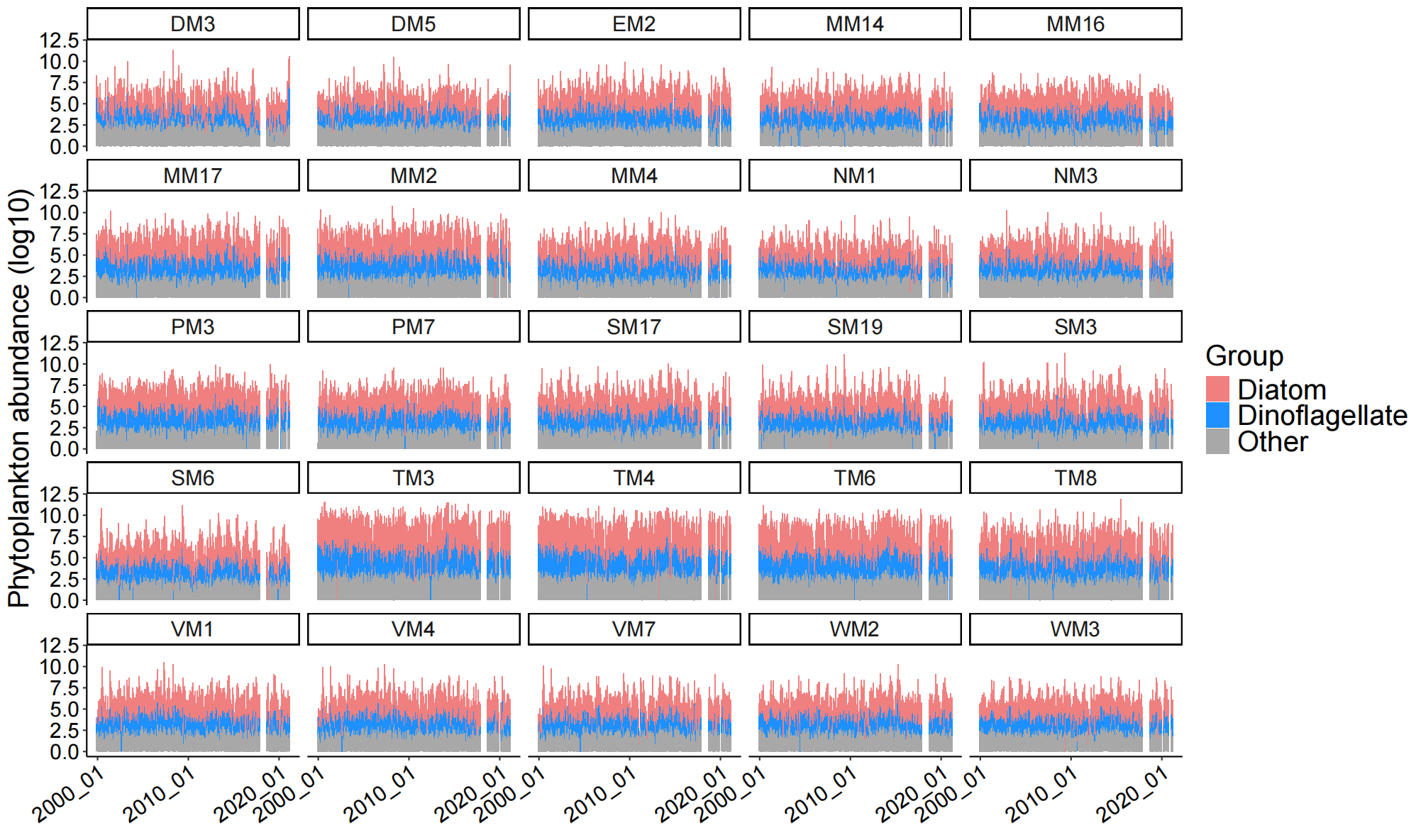


**Figure S6 | Spatiotemporal patterns (monthly) of phytoplankton absolute abundance (2000-2020).** The absolute abundance of phytoplankton (cell/mL) has been log10 transformed before plotted in the stacked bar charts. Phytoplankton groups are indicated by different colors.


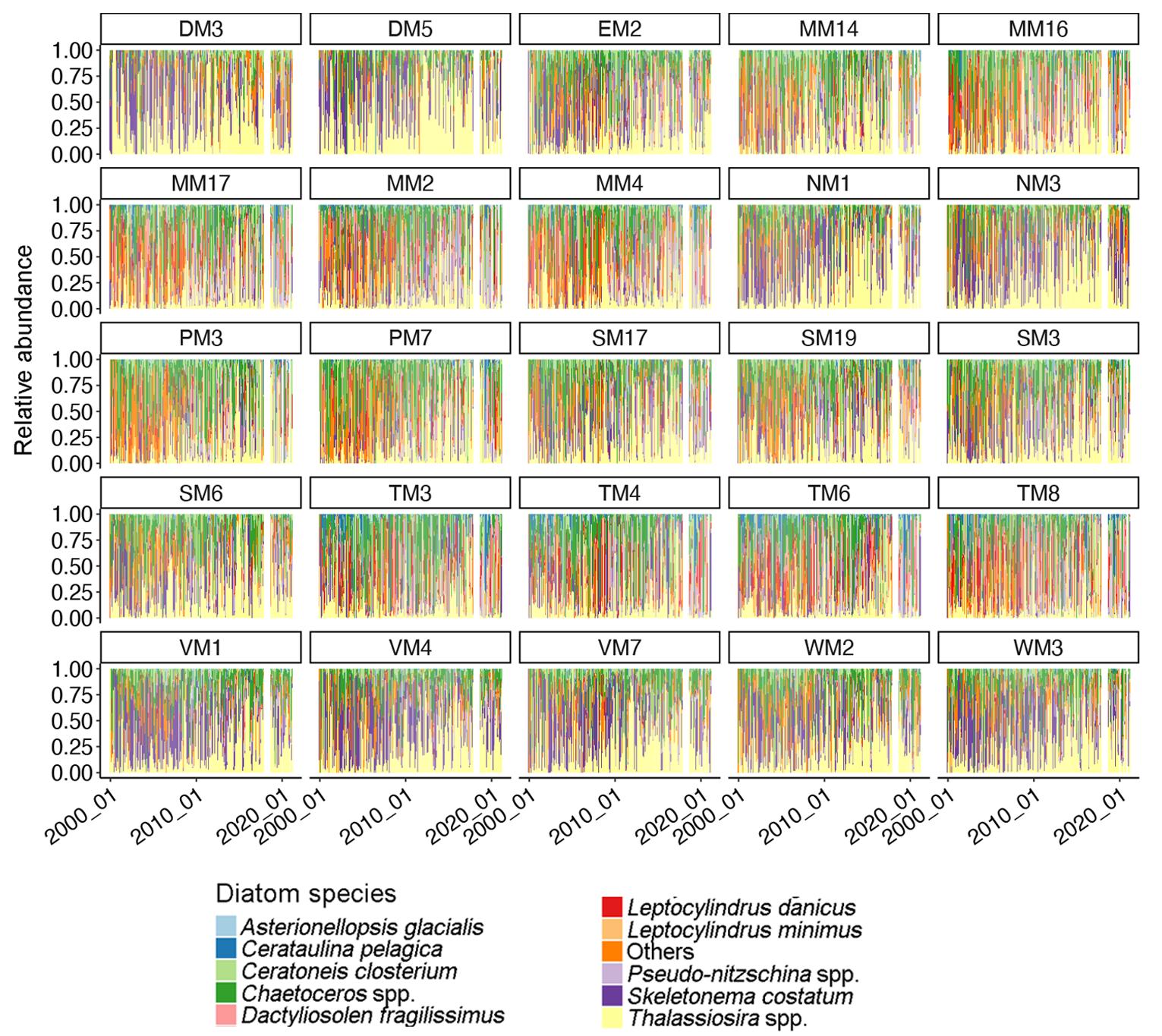


**Figure S7 | Spatiotemporal dynamics (monthly) of diatom community structures.** Relative abundances of diatoms are shown at the species or genus level (end up with “spp.”). The top 10 most abundant species (or genus) are presented whole the others were clustered into “Others”.


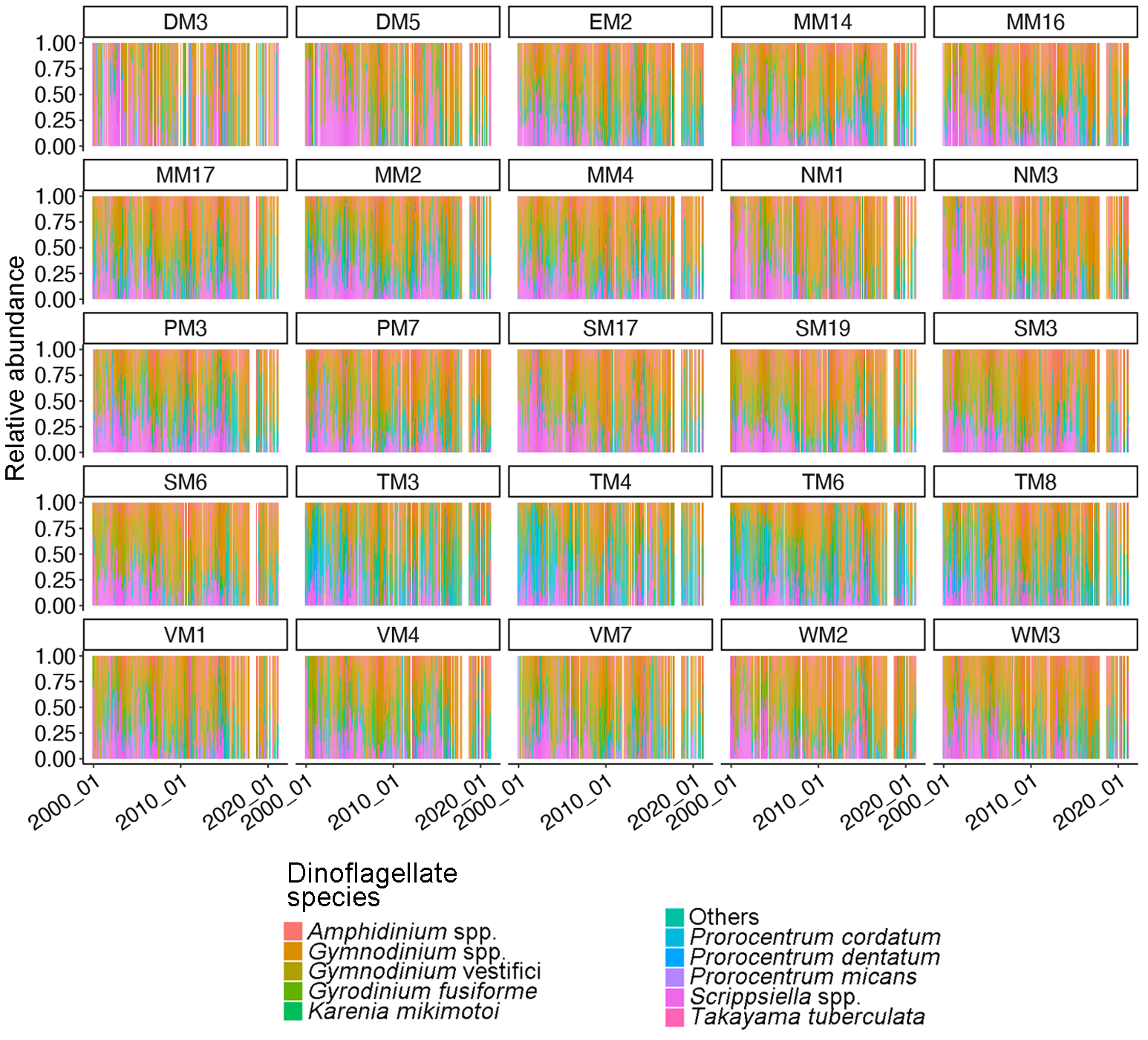


**Figure S8 | Spatiotemporal dynamics (monthly) of dinoflagellate community structures.** Relative abundances of dinoflagellates are shown at the species or genus level (end up with “spp.”). The top 10 most abundant species (or genus) were presented whole the others are clustered into “Others”.


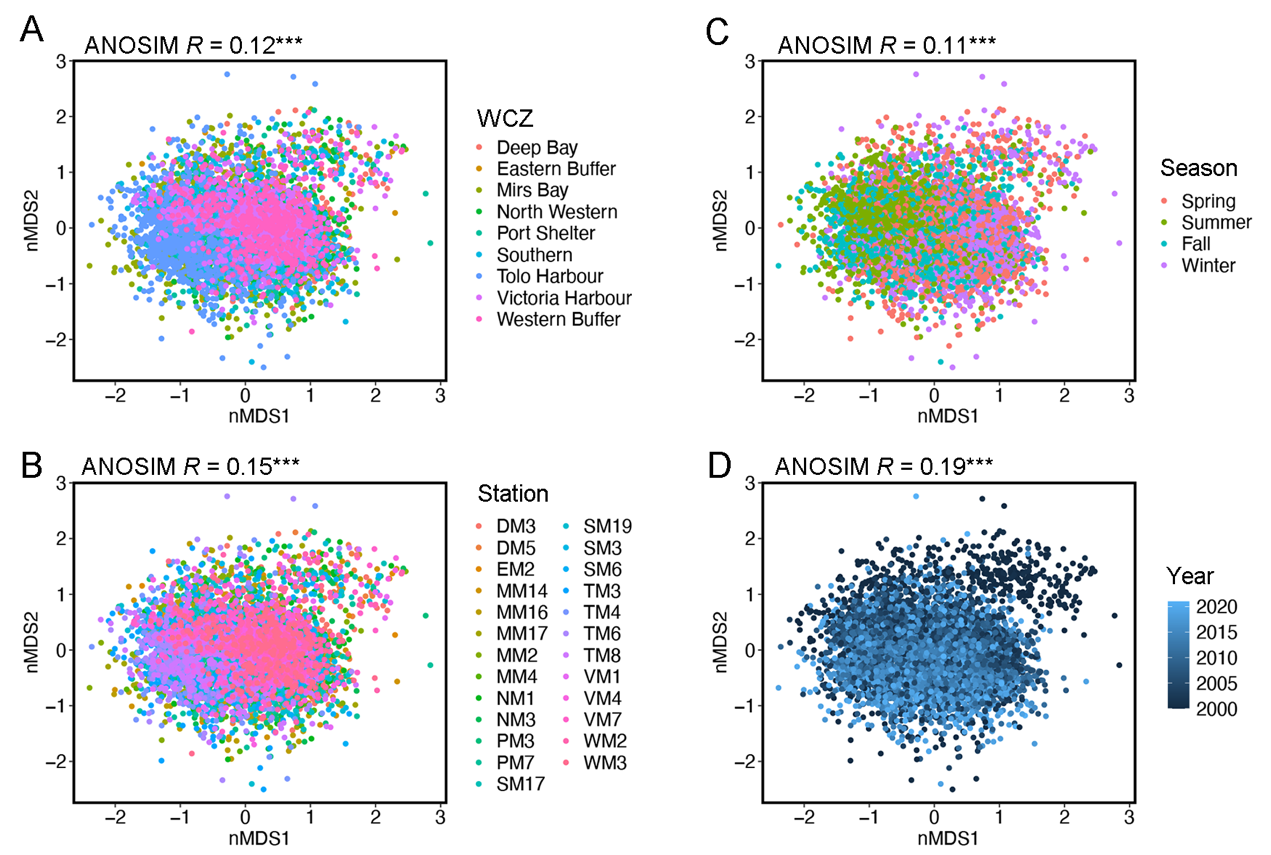


**Figure S9 | Spatiotemporal patterns of phytoplankton community structure revealed by non-metric Multidimensional scaling (nMDS).** Each dot represents a community and dots near each other mean they have similar community structures (i.e., high community similarity). Analysis of similarity (ANOSIM) tests the effects of **(A)** water control zone (WCZs), **(B)** station, **(C)** season and **(D)** year on community structure. The higher ANOSIM *R* values indicate greater effects, with statistical significance codes of *: *P* < 0.05, **: *P* < 0.01, ***: *P* < 0.001.


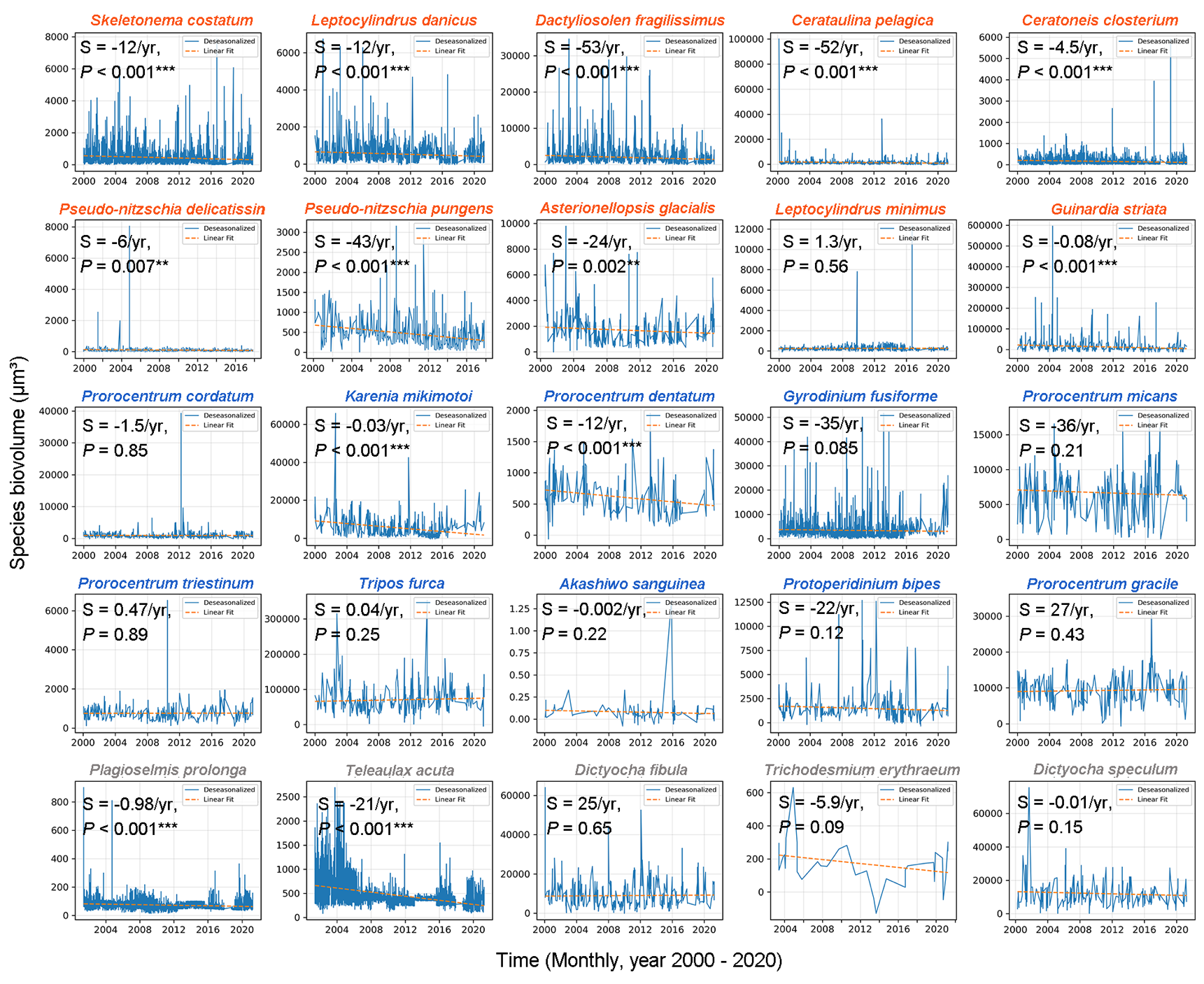


**Figure S10 | Temporal trends of species biovolume using time-series decomposition analysis.** Time-series data of species biovolume are decomposed into trend, seasonal, and residual components, using Locally Weighted Regression (LOESS, time granularity = 12 months). Blue dots in the figure represent pure trend values, with red dash line showing their linear regression (S: slope). Species are colored by groups: red for diatoms, blue for dinoflagellates, and grey for other phytoplankton groups. Significant trends are marked with codes (*: *P* < 0.05, **: *P* < 0.01, ***: *P* < 0.001).

**
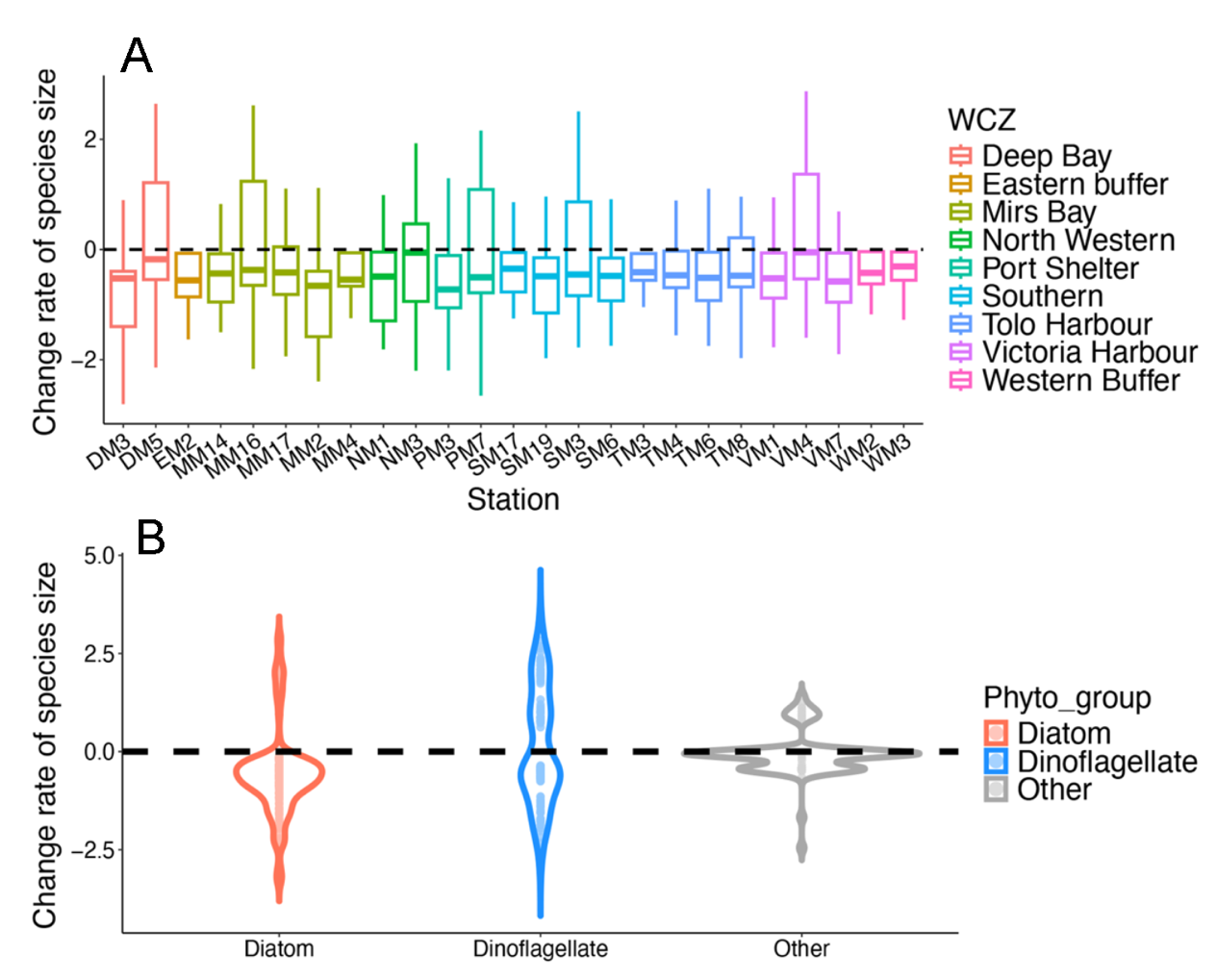
**

**Figure S11 | Temporal trends in species size at the station level.** **(A)** shows the distribution pattern of size change rates (µm³ yr⁻¹, log_10_ transformed) of all species at each station (color-coded by WCZ). **(B)** shows the phytoplankton group-level comparison of size change rates (µm³ yr⁻¹, log_10_ transformed), with values from each species at each station. Total number of counts is indicated under phytoplankton group. Only change rates with statistical significance (*P* < 0.05, linear regression) are included. The horizontal dashed line (y = 0) distinguishes negative change rates (indicating miniaturization) from positive changes.


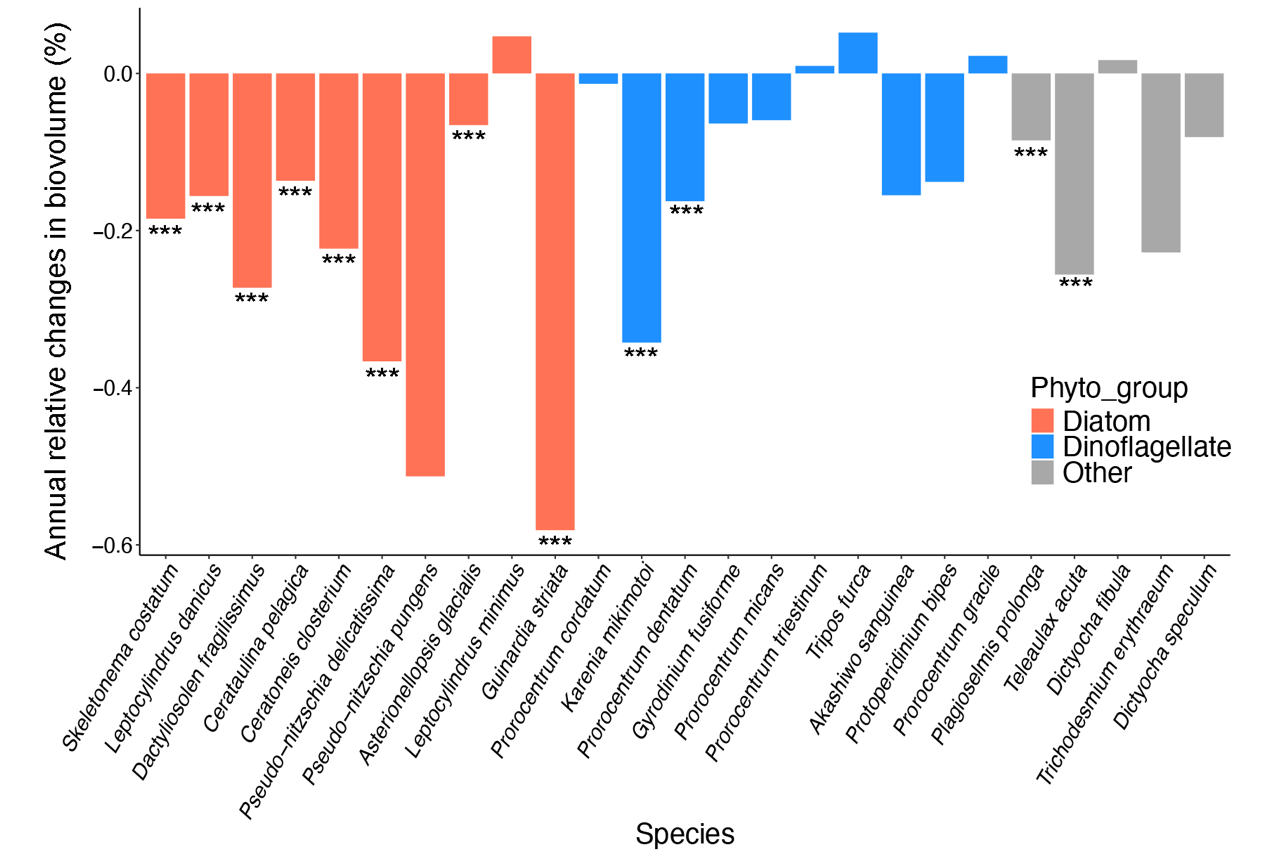


**Figure S12 | Annual relative changes in biovolume for each species.** The annual relative change in biovolume was calculated as the slope of a linear regression (lagged months vs. species biovolume) divided by the species’ initial biovolume (mean value of the first sampling year). Species are colored by phytoplankton group. Significant changes are indicated by asterisks (***: *P* < 0.001, linear regression).


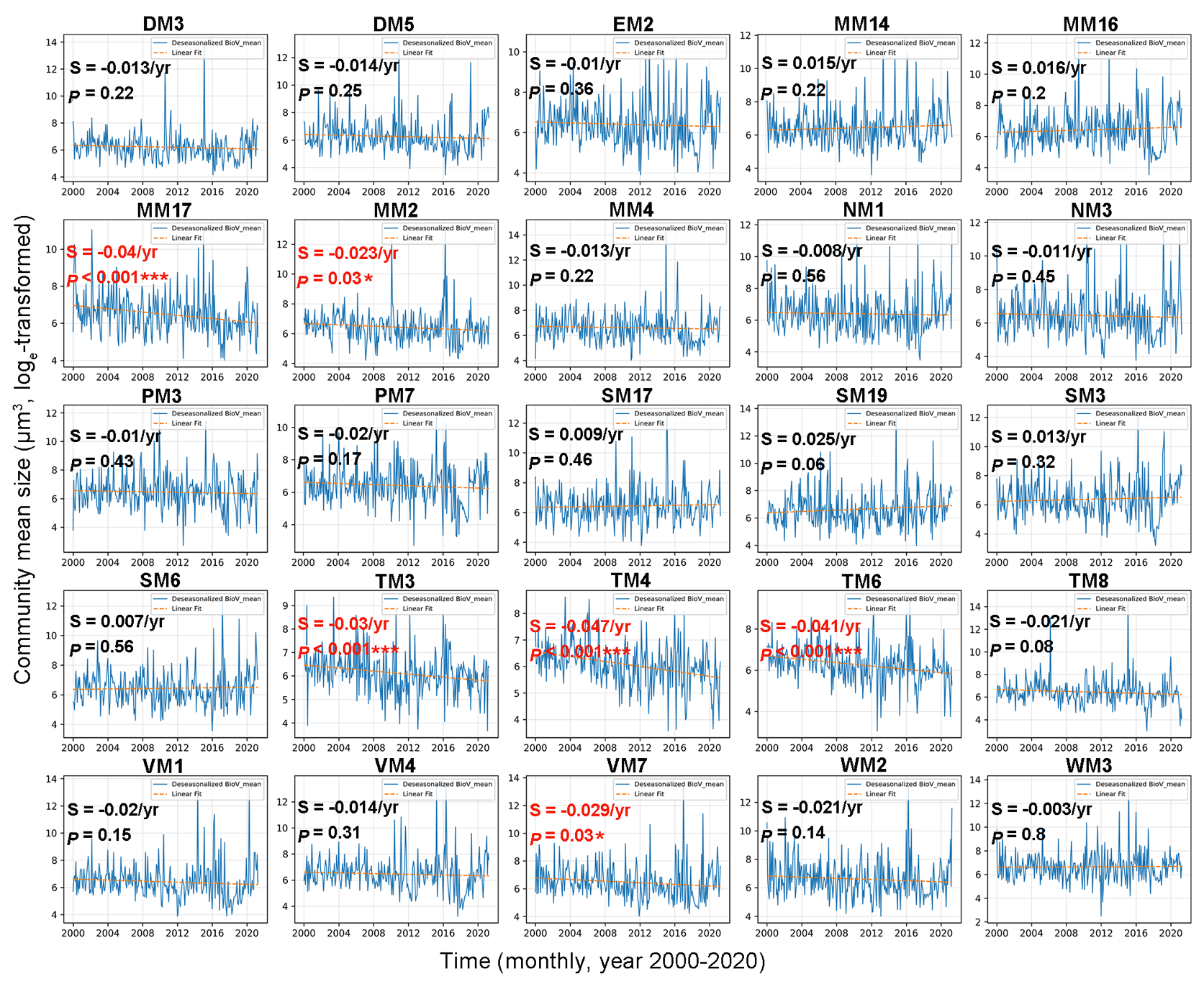


**Figure S13 | Temporal trends of community mean biovolume at each station using time-series decomposition analysis.** Time-series data of community mean biovolume are decomposed into trend, seasonal, and residual components, using Locally Weighted Regression (LOESS, time granularity = 12 months). Blue dots in the figure represent pure trend values, with red dash line showing their linear regression (S: slope). Significant trends (*P* < 0.05) were shown in red.


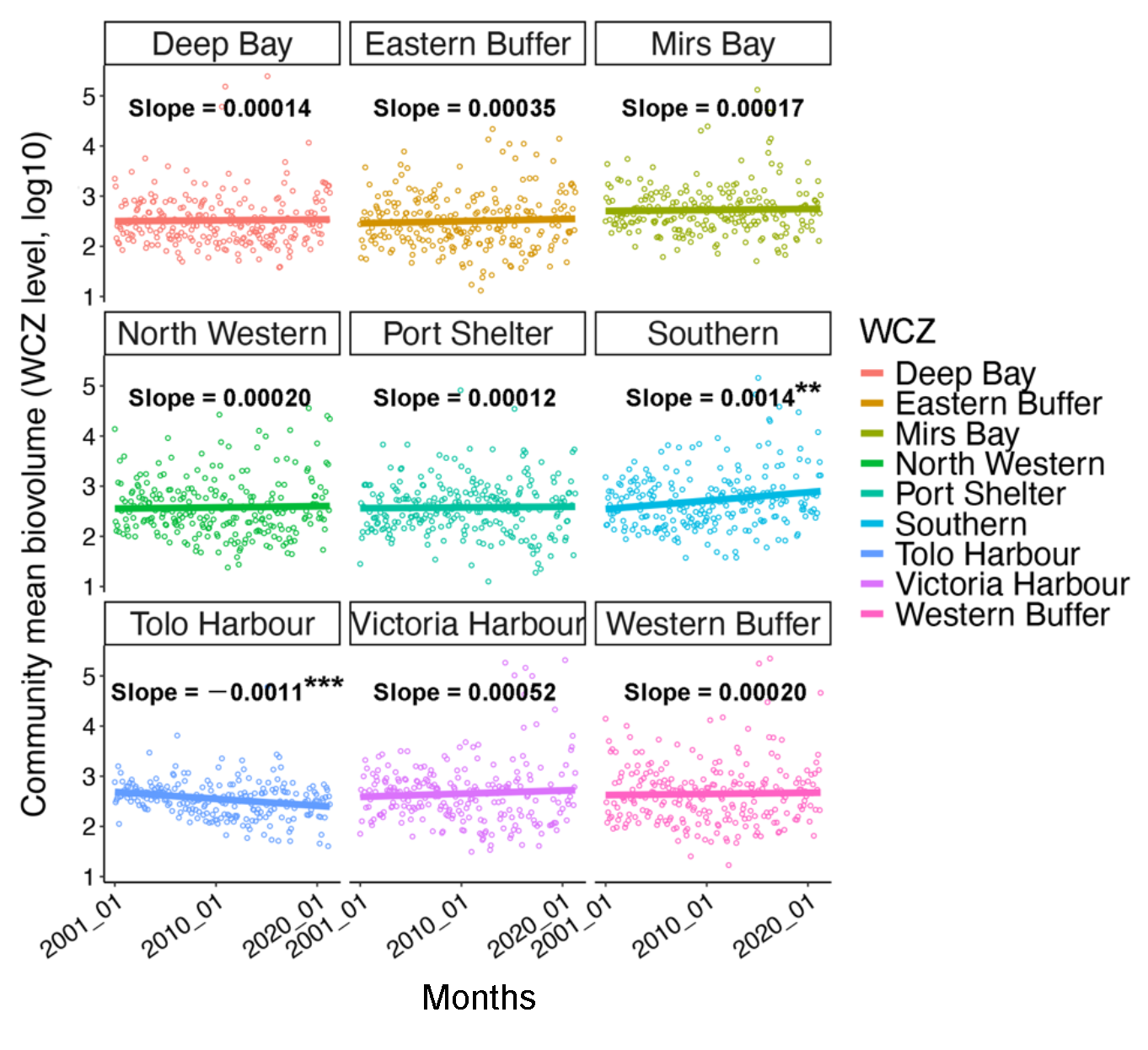


**Figure S14 | Temporal trends of community mean biovolume at the WCZ level.** Each dot represents a community mean biovolume calculated at the WCZ level (calculated by aggregating conspecific individuals across all stations within each WCZ before computing mean values). Significant negative trends, indicating community miniaturization, are marked with asterisks (*: *P* < 0.05, **: *P* < 0.01, ***: *P* < 0.001, linear regression).


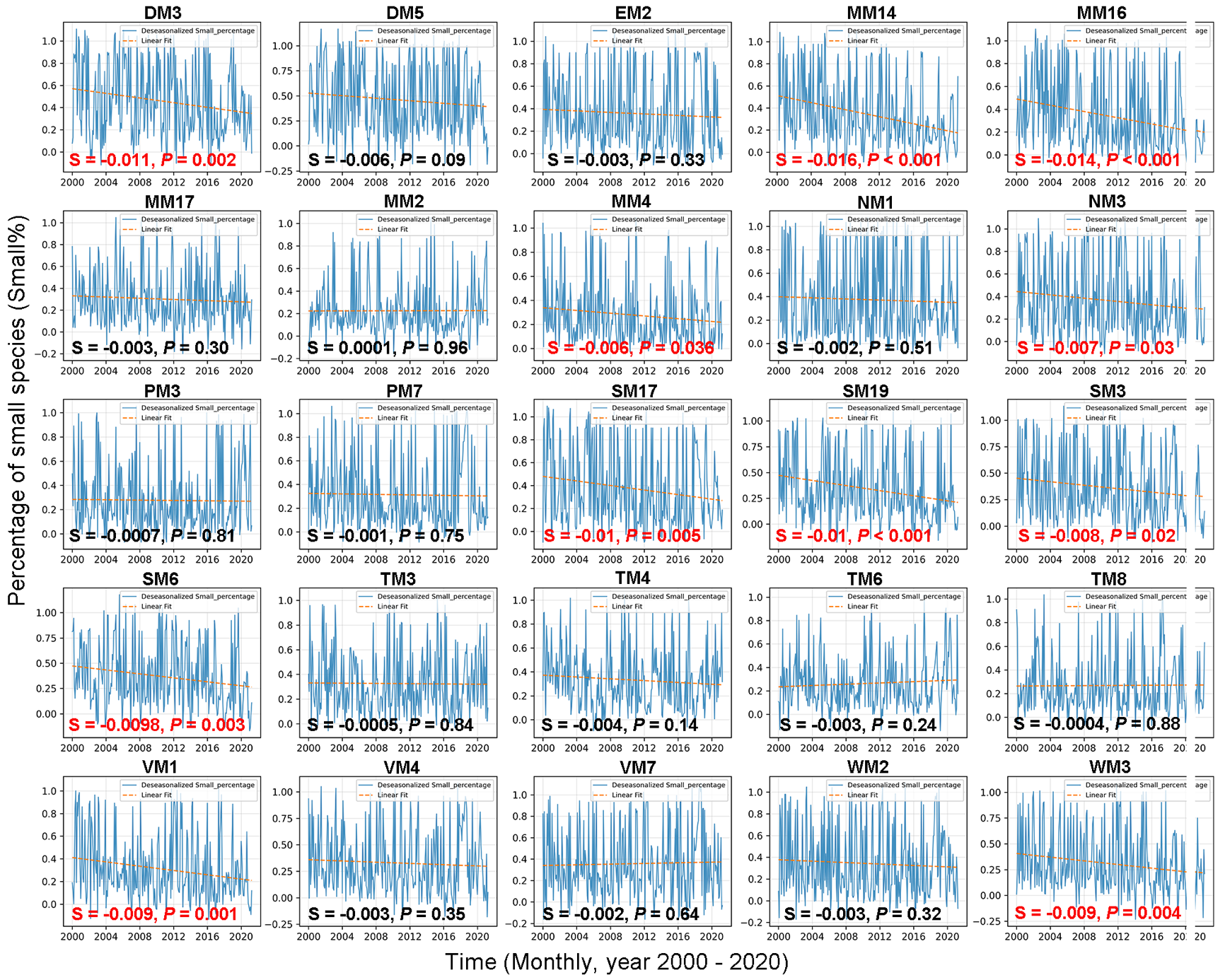


**Figure S15 | Temporal trends of percentage of small species (Small%) at each station using time-series decomposition analysis.** Time-series data of Small% are decomposed into trend, seasonal, and residual components, using Locally Weighted Regression (LOESS, time granularity = 12 months). Blue dots in the figure represent pure trend values, with red dash line showing their linear regression (S: slope). Significant trends (*P* < 0.05) were shown in red.


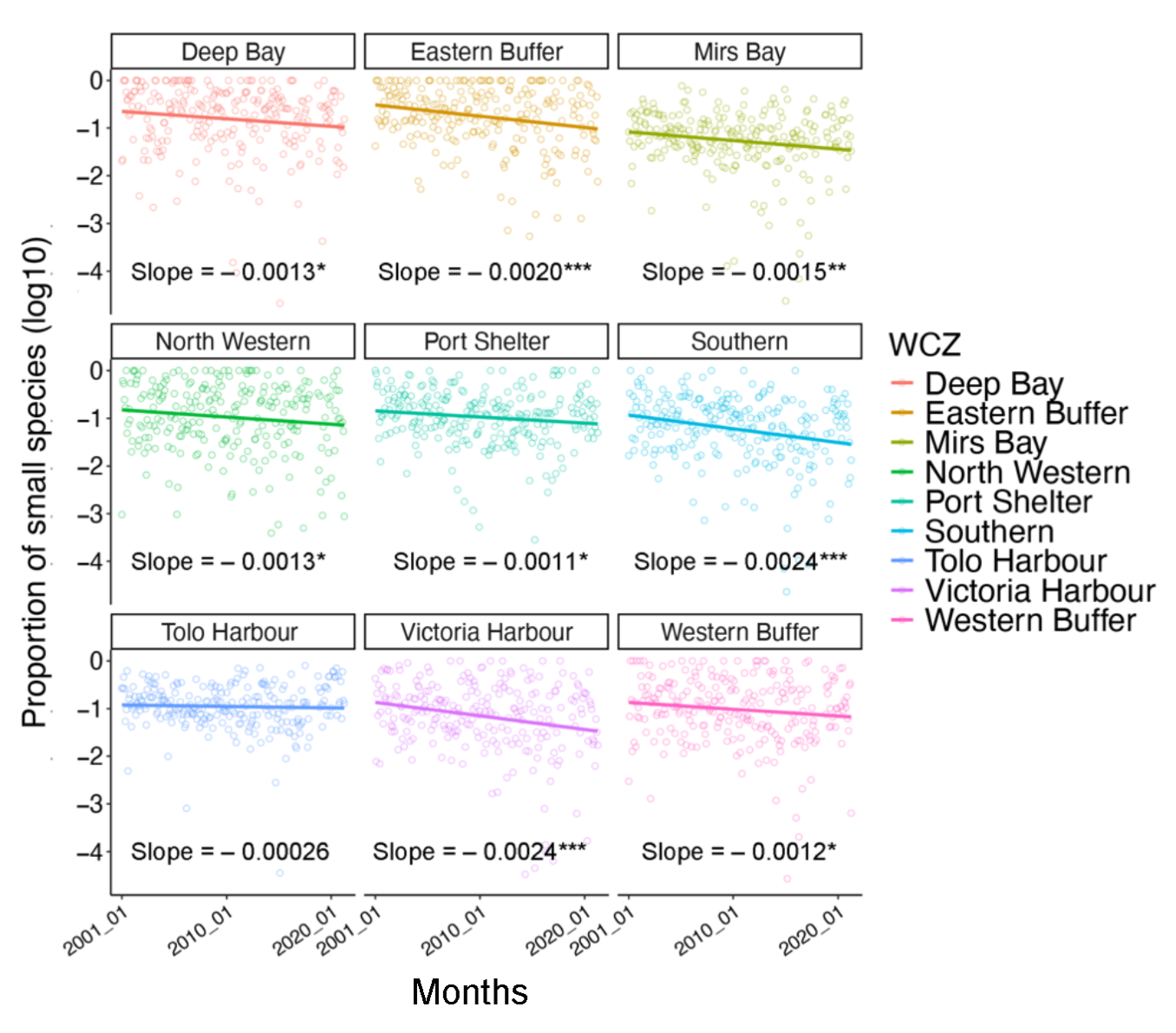


**Figure S16 | Temporal trends of Small% at the WCZ level.** Calculation follows the method described in Fig. S14. Note that Small% on y-axis is log_10_ transformed.


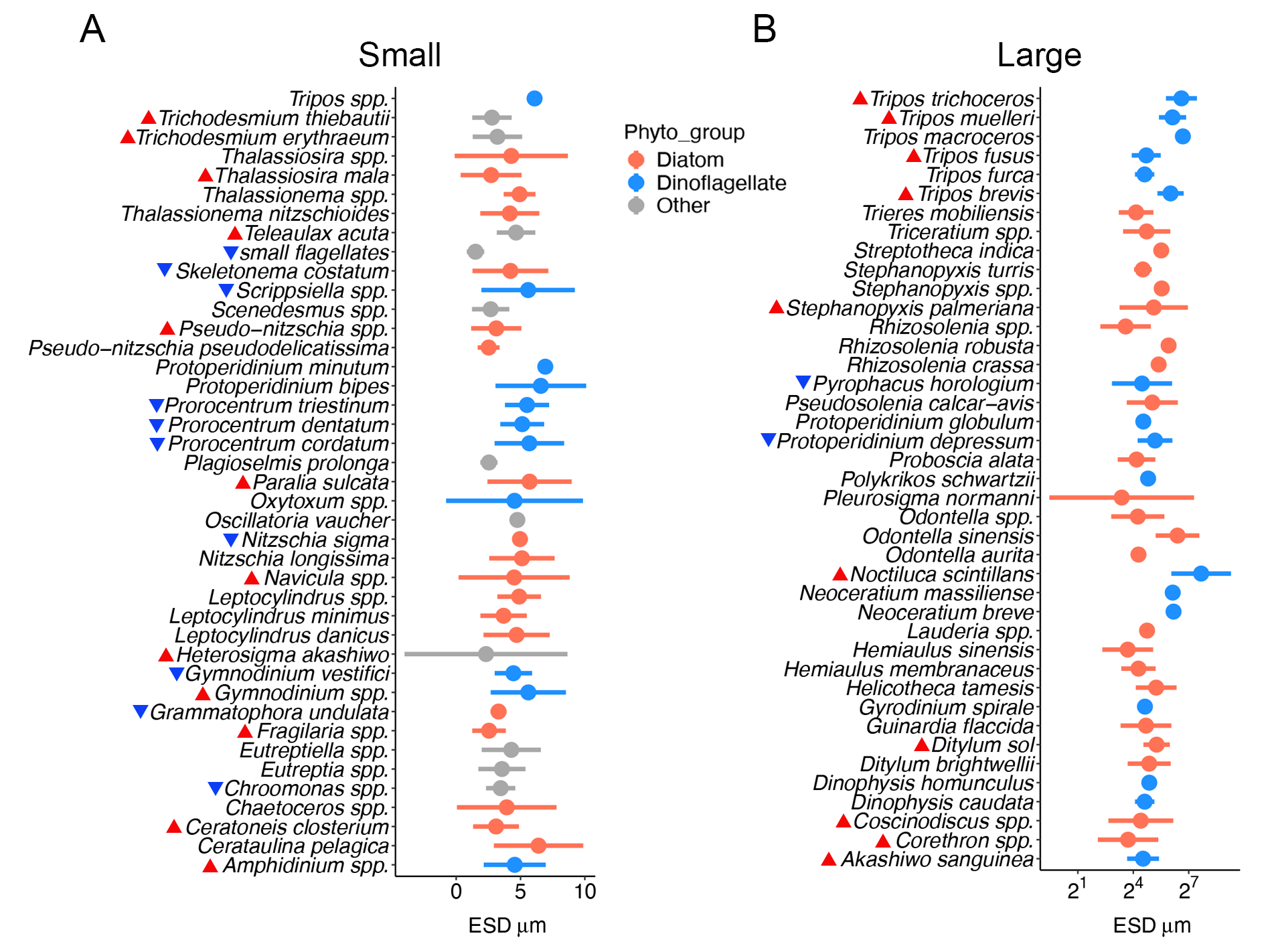


**Figure S17 | Size-class dynamics of phytoplankton communities. (A)** shows small species (lowest quartile by average biovolume across all communities) while **(B)** displays large species (highest quartile). Size is represented as equivalent spherical diameter (ESD, μm ± SD) calculated from biovolume, with colors indicating taxonomic groups. Annual trends of species relative abundance in communities are shown, with red upward triangles (▲) indicating positive trends and blue downward triangles (▼) representing negative trends (*P* < 0.05, linear regression).


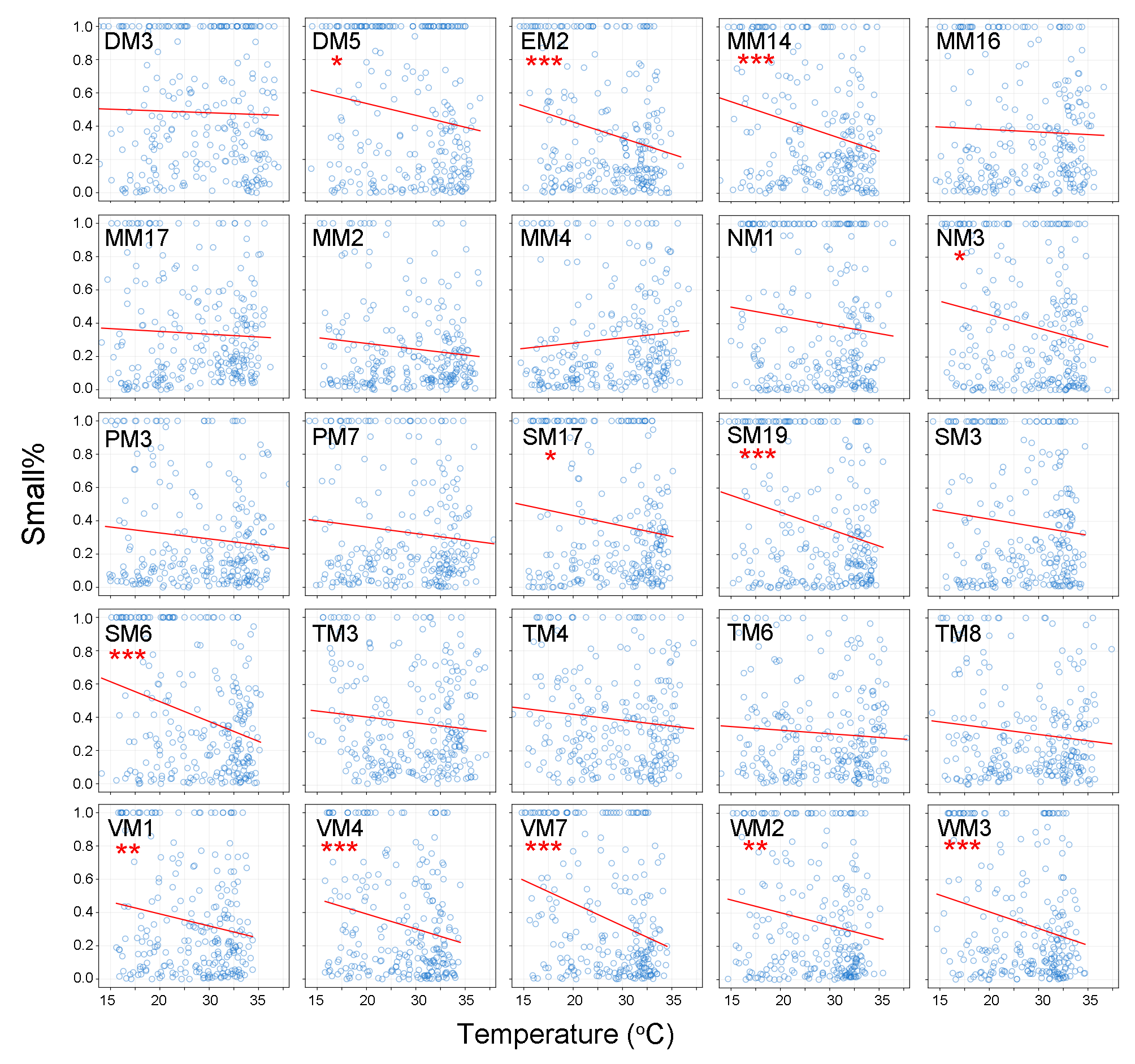


**Figure S18 | Partial effects of temperature on Small%.** Results from station-specific generalized linear mixed models (GLMMs) show partial effects of temperature on Small%, with all other environmental factors held constant. Solid red trendlines show the linear correlations with *P* values representing statistical significance (**P* < 0.05; ***P* < 0.01; ****P* < 0.001).


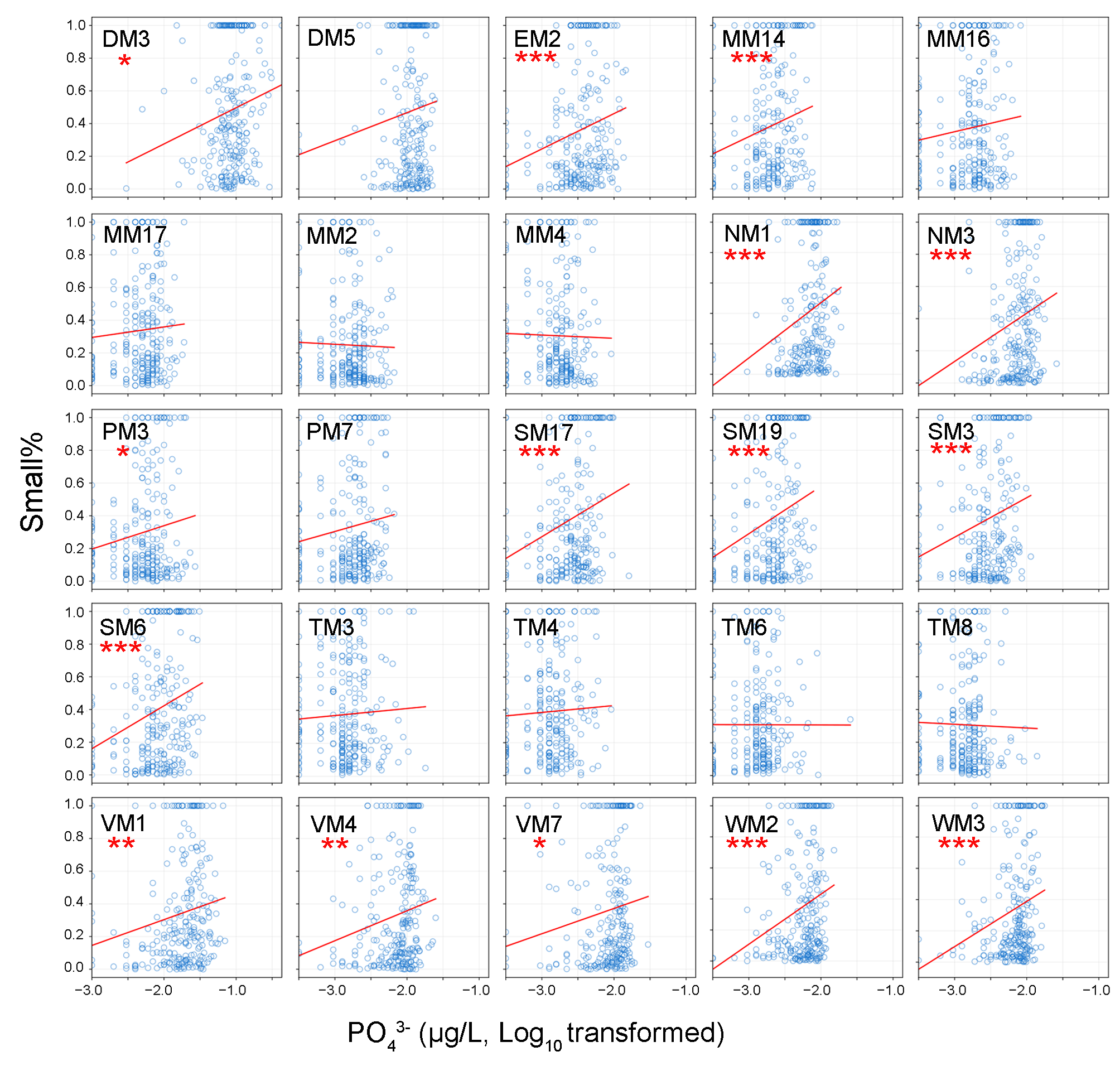


**Figure S19 | Partial effects of PO_4_^3-^ on Small%.** Results from station-specific generalized linear mixed models (GLMMs) show partial effects of PO_4_^3-^ on Small%, with all other environmental factors held constant. Solid red trendlines show the linear correlations with *P* values representing statistical significance (**P* < 0.05; ***P* < 0.01; ****P* < 0.001). Note that the scale of PO_4_^3-^ concentration at the x-axis of each station is fixed.


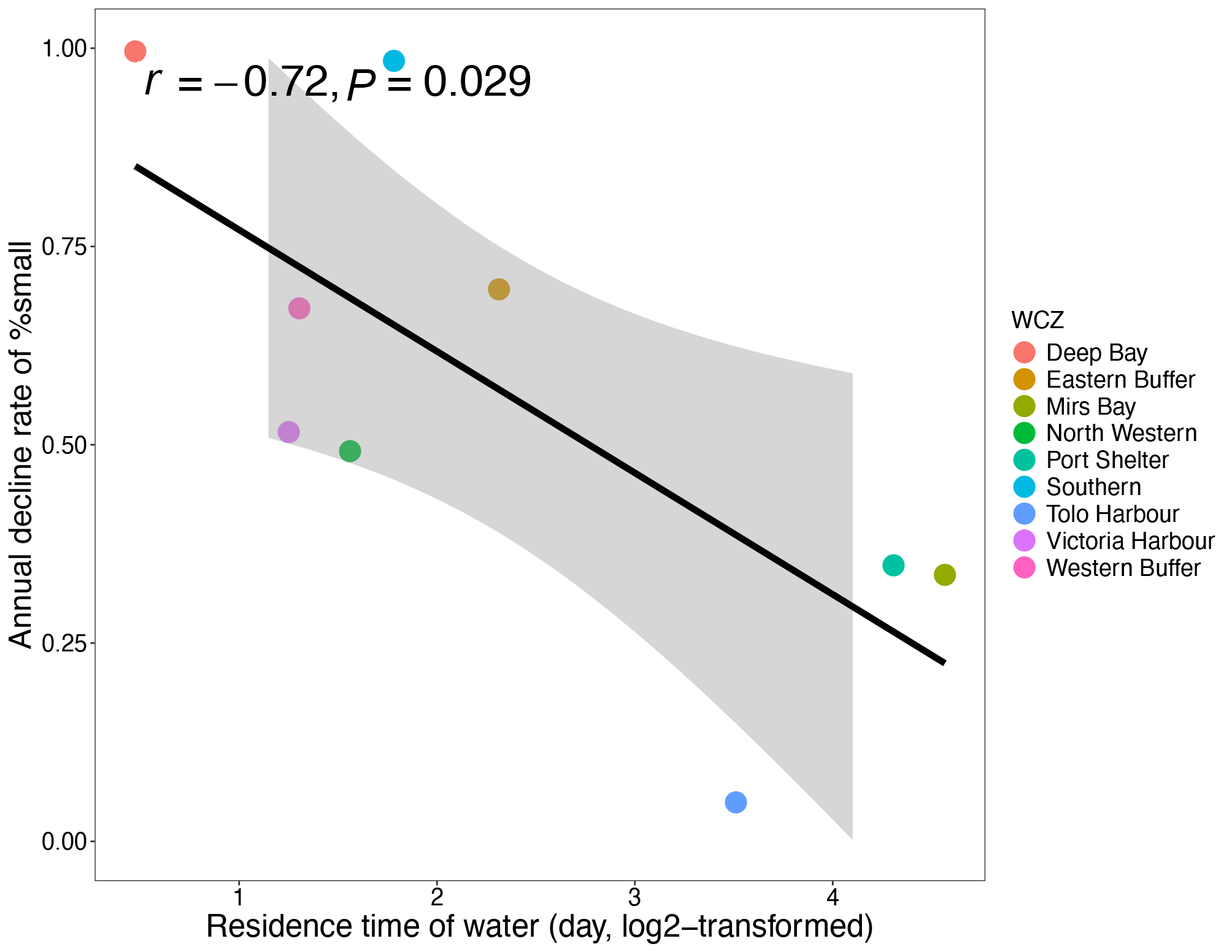


**Figure S20 | Correlation between Small% decline and residence time of water.** Tolo Harbour (blue dot) exhibits high residence time (data from Liu et al., 2023) ^7^ and low Small% decline rate (close to zero) reflecting reduced community turnover. The dashed line represents the linear regression fit, with shaded area indicating 95% confidence intervals.

**III Supplementary Tables (n = 2)**

**Table S1. Detailed information of environmental factors**

| **Abbreviation** | **Parameter** | **Unit** | **Detection Limit** | **Sampling Method** | **Ecological implication** |
| --- | --- | --- | --- | --- | --- |
| **Temperature** | Temperature | °C | 0.1 | Instrumental (thermistor), SEACAT19+ CTD and Water Quality Profiler | Physicochemical factor |
| **Salinity** | Salinity |  | 0.1 | Instrumental (electrical conductivity), SEACAT19+ CTD and Water Quality Profiler | Physicochemical factor |
| **DO** | Dissolved Oxygen | mg/L | 0.1 | SBE23Y dissolved oxygen sensor | Physicochemical factor |
| **DO_sat** | Dissolved Oxygen_saturation | % | 1 | SBE23Y dissolved oxygen sensor | Physicochemical factor |
| **pH** | pH |  | 0.1 | Instrumental (electrodemetric), SBE18 pH sensor linked to SEACAT19 + CTD and Water Quality Profiler | Physicochemical factor |
| **Turbidity** | Turbidity | NTU | 0.1 | Instrumental (nephelometric / infrared back scattering), OBS-3 turbidity sensor linked to SEACAT 19+ CTD and Water Quality Profiler | Light transparency and penetration |
| **Secchi_depth** | Secchi Disc Depth | m | 0.1 | Manual | Light transparency and penetration |
| **SS** | Suspended Solids | mg/L | 0.5 | In-house method GL-PH-23 based on APHA 22ed 2540D (weighing) | Light transparency and penetration |
| **VSS** | Volatile Suspended Solids | mg/L | 0.5 | In-house method GL-PH-23 based on APHA 22ed 2540E (weighing) | Indicating POM |
| **BOD5** | 5-day Biochemical Oxygen | mg/L | 0.1 | In-house method based on APHA 20ed 5210B | Amount of organic pollutants |
| **NH3_N** | Ammonia Nitrogen | mg/L | 0.005 | In-house method GL-IN-15 based on ASTM D3590-11 Test method B | Major nutrients |
| **NH3** | Unionised Ammonia | mg/L | 0.001 | By calculation | Major nutrients |
| **NO_2__N** | Nitrite Nitrogen | mg/L | 0.002 | In-house method GL-IN-18 based on APHA 22ed 4500-NO2 - B | Major nutrients |
| **NO_3__N** | Nitrate Nitrogen | mg/L | 0.002 | In-house method GL-IN-18 based on APHA 22ed 4500-NO3 - I | Major nutrients |
| **DIN** | Total Inorganic Nitrogen | mg/L | 0.01 | NH_3__N+NO_2__N+NO_3__N | Major nutrients |
| **Total_KN** | Total Kjeldahl Nitrogen | mg/L | 0.05 | In-house methods GL-IN-14 and GL-IN-15 based on ASTM D3590-11 Test method B | Major nutrients |
| **Total_N** | Total Nitrogen | mg/L | 0.05 | Total_KN+NO_2__N+NO_3__N | Major nutrients |
| **PO_4_^3-^** | Orthophosphate Phosphorus | mg/L | 0.002 | In-house method GL-IN-16 based on APHA 22ed 4500-P G | Major nutrients |
| **Total_P** | Total Phosphorus | mg/L | 0.02 | In-house methods GL-IN-14 and GL-IN-16 based on ASTM D515-88 Test method B and APHA 22ed 4500-P G | Major nutrients |
| **N/P ratio** | N/P ratio |  |  | Ratio of DIN: PO_4_^3-^ | Relative nutrient limitation |
| **Silica** | Silica | mg/L | 0.05 | In-house method GL-IN-17 based on APHA 22ed 4500-SiO2 F | Major nutrients |
| **Chl *a*** | Chlorophyll *a* | µg/L | 0.2 | In-house method GL-OR-34 based on APHA 20ed 10200H 2 (spectrophotometric) | Phytoplankton biomass |
| **Phaeo-pigment** | Phaeo-pigment | µg/L | 0.2 | In-house method GL-OR-34 based on APHA 20ed 10200H 2 (spectrophotometric) | Non-photosynthetic pigment |
| ***E. coli*** | *Escherichia coli* | cfu/100mL | 1 | In-house method, membrane filtration with CHROMagar Liquid *E. coli* -coliform culture | Indicating faecal pollution |
| **Faecal** | Faecal Coliforms | cfu/100mL | 1 | In-house method, membrane filtration with CHROMagar Liquid *E. coli* -coliform culture | Indicating faecal pollution |

GL: government laboratory; APHA: American Public Health Association^8^

**Table S2. Comparison of the fitness of each model**

| **Models** | **Species biovolume** | | |  | **Small%** | | |
| --- | --- | --- | --- | --- | --- | --- | --- |
|  | **MAE** | **RMSE** | ***R*^2^** |  | **MAE** | **RMSE** | ***R*^2^** |
| Ridge Regression | 0.931 | 1.191 | 0.052 |  | 0.099 | 0.120 | 0.091 |
| Random Forest | 0.767 | 0.995 | 0.339 |  | **0.094** | **0.116** | **0.153** |
| XGBoost | **0.704** | **0.933** | **0.418** |  | 0.097 | 0.119 | 0.102 |
| ANN | 0.898 | 1.149 | 0.118 |  | 0.096 | 0.117 | 0.127 |
| SVM | 0.868 | 1.144 | 0.126 |  | 0.099 | 0.122 | 0.055 |

The choice of machine (or deep) learning method in the analysis of driving factors on species biovolume and Small% was based on comparing the performances of different models on the testing data. The best model is characterized with the lowest Mean Absolute Error (MAE), lowest root-mean-square error (RMSE) and highest *R*^2^ (values in bold). ANN: artificial neural networks, SVM: support vector machine.

**IV. References**

1. Utermöhl, H.1958. Zur Vervollkommnung der quantitativen Phytoplankton-Methodik. Mitt int. Verein. theor. angew. Limnol. 9: 1-38
2. Isamu, Y., 1991. Illustrations of the Marine Plankton of Japan. 3rd edition. Hoikusha Publishing, Tokyo: 1-158.
3. Tomas, C.R. ed., 1997. Identifying marine phytoplankton. Elsevier.
4. Yang, S., Li, R. 2014. Atlas of dinoflagellates in China Seas. China Ocean Press. Beijing.
5. Law, S.P.C., Lee F.Y.K. 2013. Harmful marine microalgae in Hong Kong. Agriculture, Fisheries and Conservation Department of Hong Kong. Hong Kong.
6. Hillebrand, H., Dürselen, C.D., Kirschtel, D., Pollingher, U., Zohary, T. 1999. Biovolume calculation for pelagic and benthic microalgae. Journal of Phycology, 35(2): 403-424.
7. Liu, Y., Song, Y., & Jiao, J. J. (2023). Submarine groundwater discharge strengthens acidification in the coastal semi‐closed bays. Geophysical Research Letters, 50(17), e2023GL103788.
8. APHA, AWWA and WPCF, 1985. Standard Methods For the Examination of Water and Wastewater. American Public Health Association, Washington DC.
